# Marine Macroalgae, a Source of Natural Inhibitors of Fungal Phytopathogens

**DOI:** 10.3390/jof7121006

**Published:** 2021-11-25

**Authors:** Tânia F. L. Vicente, Marco F. L. Lemos, Rafael Félix, Patrícia Valentão, Carina Félix

**Affiliations:** 1 MARE—Marine and Environmental Sciences Centre, ESTM, Politécnico de Leiria, 2520-641 Peniche, Portugal; rafael.felix@ipleiria.pt; 2 REQUIMTE/LAQV, Laboratório de Farmacognosia, Faculdade de Farmácia, Universidade do Porto, 4050-313 Porto, Portugal; valentao@ff.up.pt

**Keywords:** algae phenols, antifungal activity, bioactive compounds, brown algae, crop losses, fungal membrane disruption, fungal resistance, lipophilic compounds, macroalgae metabolites, plant pathogens

## Abstract

Fungal phytopathogens are a growing problem all over the world; their propagation causes significant crop losses, affecting the quality of fruits and vegetables, diminishing the availability of food, leading to the loss of billions of euros every year. To control fungal diseases, the use of synthetic chemical fungicides is widely applied; these substances are, however, environmentally damaging. Marine algae, one of the richest marine sources of compounds possessing a wide range of bioactivities, present an eco-friendly alternative in the search for diverse compounds with industrial applications. The synthesis of such bioactive compounds has been recognized as part of microalgal responsiveness to stress conditions, resulting in the production of polyphenols, polysaccharides, lipophilic compounds, and terpenoids, including halogenated compounds, already described as antimicrobial agents. Furthermore, many studies, in vitro or in planta, have demonstrated the inhibitory activity of these compounds with respect to fungal phytopathogens. This review aims to gather the maximum of information addressing macroalgae extracts with potential inhibition against fungal phytopathogens, including the best inhibitory results, while presenting some already reported mechanisms of action.

## 1. Introduction

Plant pests pose a paramount problem that has been increasing in recent years. The exact production losses due to these phytopathogens are hard to quantify but it is estimated that plant pests account for 20–40% of annual crop production losses [1,2], at a cost of more than 185 billion euros [3]. Included among these pests, fungal pathogens are one of the most damaging agents in plants, accounting for the devastation of myriad fruits and crops, which results in vast economic losses [4], and ultimately reduces food availability for a continuously increasing world population [5,6]. In fact, diseases provoked by fungi or related microorganisms have already caused starvation scenarios, such as the Irish Potato Famine in the 19th century, caused by a fungal-like oomycete, which led to a million of deaths, mass emigration, and economical and political crisis in Ireland [7,8]. Phytopathogenic fungi were also responsible for the baring of landscapes caused by Dutch elm blight and chestnut blight [8] and the complete ruin of 30% of world food crops in 2012 [3]. Currently, it is predicted that phytopathogenic fungi are responsible for about 80% of plant diseases [9,10,11], for which the absence of control can lead to disastrous global crop losses [6,12]. Even the remaining crops, potentially infected but without symptoms, can raise concerns about consumption safety [13]. Moreover, current and forecasted climatic change scenarios, leading to the increase of temperature and humidity, are crucial conditions promoting the dispersion and development of phytopathogenic fungi, giving cause for extra concerns [12,14].

The regular application of agrochemicals with antimicrobial properties is the most effective method against these microbial phytopathogens, but it is expensive and environmentally harmful, prevailing in the ecosystem and damaging it [15,16]. Every year, farmers spend more than 6 billion euros on such products to control the microbial infections, which represents a quarter of the costs for agricultural purposes [17]. For sustainability reasons, novel alternative methods have been sought that will have the same effectiveness, improve agricultural techniques, and enhance food production, ensuring the quality and security of food [18]. Several techniques and methodologies have been tested to minimize plant and financial losses either by directly targeting the microbial phytopathogens or by preventive measures, conferring resistance to the plant hosts. The laboratory manipulation of synthetic compounds to increase the effectiveness of products [19] or the introduction of “site-specific fungicides” [20] to control the most problematic and common microbial pathogens, have been suggested. Nevertheless, these products remain inefficient due to the great genetic resources and adaptative abilities of phytopathogens, which allow them to acquire resistance and overcome the efficiency of these types of products [20,21]. The biocontrol technique, characterized by the introduction of an antagonist microbial organism, harmless to the host but damaging for the phytopathogen [14], has been tested in vitro [2,22,23,24,25,26] and shown a great potential in field applications [2]. This methodology is characterized by the absence of chemicals, providing a viable and sustainable agriculture [27]. Although some limitations associated with the establishment and maintenance of biocontrol agents have been identified [2], including their interaction with the plant microbial community [28], the continuous stress conditions provoked in the host plant, the inconsistent results among tests [14,29,30], and the poor effectiveness compared to chemical fungicides, are factors which could and should be improved [29,30,31]. Though their potential can be enhanced through their combination with chemical interventions [28,32], this fails to solve the harm these compounds pose to the environment. The exploitation of genetic manipulation to alter the plant host genome with the insertion of resistance genes [33] was quickly shown to be ineffective against non-target phytopathogenic microorganisms and/or the emergence of new microbial races [15]. Therefore, the continuous search for biodegradable natural compounds, eco-friendly and effective against phytopathogenic microorganisms, is paramount [34], promising as it does to enhance food production and ensure the quality and security of agricultural products [18].

Marine habitats have been increasingly investigated due to the potential of bioactive products synthesized by the micro- and macro-organisms inhabiting them [35] being used in medicine and industry [36]. Seaweeds are one of the most attractive sources of bioactive substances due to their unique and diversified production of phenolic compounds, polysaccharides, fatty acids, and pigments. It is known that macroalgal applications have the potential to go beyond the ongoing uses in cosmetics, agricultural fertilizers, and the food industry [37]. Marine algae have revealed interesting compounds with antibiotic activity against pathogenic bacteria and fungi. Polysaccharides, polyphenols, carotenoids, proteins, peptides, sterols, terpenes, and fatty acids, among others, are the main constituents of algae that are associated with the antimicrobial properties of seaweed extracts [38,39,40]. Moreover, some of these algae compounds are capable of stimulating the natural defences of plants and promoting their resistance against microbial attacks, exhibiting a priming potential [39,41].

Considering the problems referred to above and the constant reduction of the effectiveness of available eco-friendly methodologies, given the promising results of in vitro assays, macroalgae constitute a source of diverse and natural compounds with antimicrobial potential against phytopathogenic fungi. Given this framework, the present review focuses on the potential of macroalgae-derived products, aiming to combine the available information regarding the potential/activity of fungal phytopathogen inhibition, while trying to clarify/link some “compound mode-of-action” and provide help and insights for future research into antimicrobial products derived from seaweeds.

## 2. Materials and Methods

For the present literature revision, a search was performed in the SCOPUS database to retrieve the maximum amount of information about the antimicrobial potential and activity of macroalgae available up until 25 February 2021. The following word combinations were used: (Antifung* OR fungicid*) AND (Plant* OR crop* OR agricultur* OR veget* OR phytopatho*) AND (Macroalga* OR seaweed). The search returned 126 documents.

## 3. Macroalgae Potential in the Eradication of Fungal Infections in Plants

### 3.1. Phytopathogenic Fungi

Fungal phytopathogens represent a significant threat for plant species [9,42], colonizing a wide range of diversified host plants. Their infections are particularly worrisome in crops for human consumption [42] because they can limit the availability of food to satisfy human nutritional needs. Strange and Scott already highlighted this problem in their review of 2005 [6] describing all the fungal pathogens and respective diseases from the main crop plants used for consumption. Specifically, fungi exhibited a devastating effect on cereal crops (maize, wheat, soybean, barley, millet, and rice), fruits (including a vast range of plant species), roots, tubers (yam, potato, and sweet potato), and vegetables [6,42]. The damages caused to a given plant depend upon the fungal feeding requirement [10]. The biotrophic fungi completely rely on their living host to survive and to grow [43]. Nevertheless, the fast reproduction of the fungi leads to a propagation not sustained by the plant, resulting in deformations of the host shape in various organs and the ripping of superficial tissues, leaving the plant susceptible to other pathogens and diseases. Necrotrophic fungi colonize the dead plant host, and their attack can also happen in various organs [44], affecting the superficial tissues of roots and trunk, as well as the inner vessels of the plants [14]. Hemibiotrophs are fungi that require the host to be alive, and, later on, they need dead matter to complete their life cycle. The damage caused by this type of fungi is local and specific [43,45]. Several researchers have been trying to compile information about phytopathogenic fungi, including the generation of databases analysing the molecular interactions between host and pathogen, such as the “One Stop Shop Fungi” [46] and projects aiming at the collection of phytopathogenic genera reported in the literature [47,48,49], as well as the “Genera of phytopathogenic fungi: GOPHY” project developed in 2017. This project has already described hundreds of species distributed across 62 genera. Table 1 presents some of the most relevant phytopathogenic fungal genera, as well as their respective targets (host plants).

These phytopathogenic microorganisms are an old and recurrent problem that has been extensively studied to find effective solutions to control their worldwide propagation. A promising alternative based on natural compounds of macroalgae (direct use of dry powder or extracts) has been explored since the last century, testing the antifungal potential of metabolites through in vitro methodologies (e.g., mycelial and spore germination inhibition) and in vivo assays (e.g., validation in plants). The antifungal potential of extracts obtained from macroalgae is highly influenced by the methodology and solvents used to obtain them, which promote the extraction of different types of compounds with different bioactivities. Several researchers highlight the use of organic solvents as the most promising way to obtain extracts with antifungal activity in macroalgae [50,51], which can be ascribed to their high affinity for phenolic and lipidic compounds, both of which are associated with good inhibitory activity against fungi [52]. The most reported mechanism for this antifungal activity is the disruption of the fungal membrane caused by bioactive algae extracts [53], which disturbs the electron transport chain, increasing membrane fluidity and causing conformational disorders that are expressed by the outflow of important cytoplasmatic components [54,55], resulting in fungal cell death [56].

**Table 1:** Relevant phytopathogenic fungi genera and their hosts.

Fungal Genera	Host Plant	References
*Alternaria*	Fruit plants, such as tomato (*Lycopersicon esculentum*) and apple (*Malus domestica)*	[49,57,58,59]
*Aspergillus*	Seeds, nuts, and fruits of a wide range of plant species	[57,58,60,61,62]
*Botrytis*	Wide range of plant hosts	[57,63,64]
*Colletotrichum*	Mediterranean plants and trees (fruits), tropical species and vegetables	[42,47,65,66,67,68,69]
*Fusarium*	The broad range of hosts include mono- and dicotyledons in greenhouses, cereals crops, and other plant species, such as tomato, upland cotton (*Gossypium hirsutum*), banana (*Musa* sp.), and plants belonging to the Brassicaceae family	[42,52,57,63,64,70,71,72,73,74,75]
*Penicillium*	Fruits and vegetables	[57,58,76,77]
*Puccinia*	Wheat crops (*Triticum aestivum*)	[42,47,64,78]
*Rhizoctonia*	Root pathogen of a wide range of hosts, including tomato, soybean (*Glycine max*), pepper (*Capsicum annuum*), eggplant (*Solanum melongena*), watermelon (*Citrullus lanatus*), upland cotton, sunflower (*Helianthus annuus*), rice (*Oryza sativa*), and potato *(Solanum tuberosum*)	[32,57,71,72,73,74,75,79,80]
*Rhizopus*	Brassicaceae plants	[57,70]

### 3.2. Macroalgae Potential against Phytopathogenic Fungi

#### 3.2.1. In vitro Antifungal Potential

The potential of activities presented by the metabolites produced by seaweed is influenced by a myriad of combined environmental [81,82] and biological [83,84,85] factors of the algae species involved, in addition to the methodology adopted for the recovery of the diverse bioactive compounds [85,86,87,88,89,90,91]. The antifungal potential/activity of the macroalgae follows the same pattern.

An overwhelming majority of studies reporting antifungal activity/potential come from brown algae, followed by the green and red algae (extensively reported in the Supplementary Material; Tables S1–S12). Additionally, there are studies demonstrating an exclusive antifungal activity from brown macroalgae against fungi species (Table 2). *Botrytis cinerea* [63], *Cladosporium herbarum* [56], *Geotrichum* sp. [63], *Phialophora cinerescens*, *Phoma tracheiphila* [65], *Sclerotinia sclerotiorum, Sclerotium rolfsii* [92], and *Verticillium dahliae* [63,93] are some examples of fungi that only presented susceptibility to algae extracts belonging to the class Phaeophyceae. Exceptions were found in the species *Colletotrichum gloeosporioides*, *Pseudocercospora fijiensis* [94], and *Pyricularia oryzae* [95], which were only inhibited by red algae, a group also possessing a large amount of diverse relevant compounds [96]. The genus *Alternaria* is one of the most prevalent phytopathogenic groups, responsible for soft-rotting infections and *Alternaria* blight in apple trees and tomato plants, respectively, leading to important fruit losses [58,97]. In addition to this genus, *Penicillium expansum* and *Aspergillus niger* are also soft-rotting devastating fungi for a large range of fruits and vegetables. In a work performed by Vehapi, the in vitro antifungal potential of a green alga, *Ulva lactuca*, was demonstrated, suggesting the presence of polyphenols responsible for the oxidation of important elements present in *Alternaria alternata* and *P. expansum* [58].

*Colletotrichum* is one of the most devastating genera of phytopathogenic fungi, due to its cross-infection capacity affecting a large range of hosts, including fruit trees (tropical and Mediterranean species), vegetables, and one of the most economically important plants, sugarcane [42,47,65,66,67,68,69]. The enormous losses caused in strawberry cultures are noticeable [98]. Moreau and colleagues reported significant inhibitory activity exhibited by hexane extracts of brown algae, *Dictyota dichotoma* and *Dilophus spiralis*, against *Colletotrichum acutatum* [65]. This species can damage the fruit (black spot) and root (necrosis and crown rot) of strawberry, pepper, eggplant, tomato, and beans. Additionally, *Colletotrichum falcatum*, a causative agent of red rot in sugarcane, is responsible for losses of hundreds of million dollars every year [99,100]. Ambika and Sujatha [66] tested the susceptibility of this fungus to the aqueous and ethanolic extracts of *Sargassum myricocystum*, *Gracilaria edulis*, and *Caulerpa racemosa*, and observed higher antifungal activity in brown algae, corroborating their higher potential. The ethanolic solvent used promoted the extraction of lipophilic compounds from macroalgae that are known for their antifungal activity. Also present in brown algae is a subgroup of phenolic compounds, the flavonoids, possessing a wide range of bioactivities, antifungal activity among them [66]. Rhodophyta algae also exhibited antifungal activity against the agents responsible for anthracnose, *Colletotrichum* species, in tropical crops [67] and *Capsicum annuum* plants [69]. The high inhibition of red algae observed against *C. gloeosporioides* and *Colletotrichum musae* can be related to the natural compounds produced by algae as a defence mechanism against microbial attack [101,102]. The sessile characteristic of the algae leads to the production of phenols [103] and terpenes (di-, sesquiterpenes) [102], including halogenated monoterpenes, [101] to self-protect under stress conditions [68], and other compounds, such as fatty acids [104], to which can be attributed antifungal activity against phytopathogenic fungi [102]. Moreover, Mani and Nagarathnam demonstrated the capacity of ƙ-carrageenan, a polysaccharide produced by the Rhodophyta group, to alter the membrane permeability of *C*. *gloeosporioides*, an antifungal mechanism that can suppress their development [69].

The genus *Fusarium* is the most devastating soil-borne agent for several crops, and is known to produce toxins that are prejudicial for animals and in plants to be responsible for fusarium wilting, snow mold, the whitening of ears in crops, and root rot diseases [52,57]. Although the majority of studies focus on the evaluation of algae extracts as antifungal agents against two persistent phytopathogenic species, *Fusarium oxysporum* and *Fusarium solani* (Table 2), which are involved in vascular bundle wilt with incidence in various economically relevant plants, such as eggplant, watermelon [72], pigeon pea [105], sunflower, and tomato [75], there are also a high number of studies reporting the potential of algae extracts tested against a wide range of other *Fusarium* species [51,57,63,96].

Diverse macroalgae species belonging to red, green, and brown macroalgae have been investigated for their antifungal potential against *Fusarium* species, and their potential has been observed in in vitro assays, as well as in field and in greenhouse conditions [71]. Rizvi and Shameel reported a higher susceptibility to methanolic extracts produced by Chlorophyta, Phaeophyta, and Rhodophyta in *F. solani*, while *F. moniliforme* was only inhibited by methanolic extracts from brown and red alga, *Dictyota hauckiana* and *Botryocladia leptopoda*, respectively, showing a different interaction between extracts and fungal species [96]. In another work, Tyśkiewicz and colleagues presented the antifungal activity of an aqueous extract, obtained by supercritical carbon dioxide extraction from *Fucus vesiculosus*, as a potential antifungal agent and/or fungistatic due to the complete degradation of macroconidia of *F. oxysporum* and *F. culmorum* [57] observed in in vitro tests. Such results are extremely important since these globally spread species are very persistent in soil, making their elimination much more challenging.

Malini [51] tested different promising organic solvents to extract bioactive compounds possessing antimicrobial activity. Their antifungal potential was confirmed, and all the organic extracts of *Anthophycus longifolius* (then identified as *Sargassum longifolium*) were able to inhibit the growth of *Fusarium* sp., chloroform highlighted as the most effective solvent [51]. A diversified range of different compounds was identified in this extract, namely proteins, phenolic compounds, alkaloids, coumarin, and sugars [51]. Some of these compounds, such as phenolic compounds, in addition to terpenoids, a class of organic compounds usually abundant in brown algae, are commonly reported to possess antifungal activity [63] against phytopathogenic fungi belonging to the *Fusarium* genus [106]. Additionally, the high antifungal activity of the chloroform extract of *Hormophysa cuneiformis* and the methanolic extract of *Polycladia myrica* (then named as *Cystoseira myrica*) and *Sargassum cinereum* against *Fusarium* spp. have been associated with their richness in fatty acids, including saturated (lauric acid, palmitic, myristic, and stearic), monounsaturated, and polyunsaturated fatty acids (arachidonic, dihomo-γ-linolenic, and cis-11,14-eicosadienoic) [56], as well as to the presence of essential oils with antimicrobial activities already described [107]. Specifically, some of these acid compounds were tested against *Fusarium* spp., and lauric, myristic, and palmitic acids demonstrated moderate inhibitory activity [108]. In the study of Ambreen et al. [109], the presence of polyunsaturated esters was found to be responsible for the antifungal activity of an ethanolic extract of *Sargassum ilicifolium* against *F. oxysporum* by disrupting its membrane.

In parallel with the *Fusarium* genus, several studies have been developed to combat the propagation of phytopathogenic *Macrophomina phaseolina* [110], since this species is known to cause significant damages in food crops, including plants used in human diets [14,52,111,112]. Khan and colleagues found a general inhibitory activity against this species in the extracts of green, brown, and red algae [52]. However, a higher activity from the aqueous and methanolic extracts obtained from *Sargassum tenerrinum* was registered. Despite the common existence of some differences between algae species from the same genus [52], *Sargassum ilicifolium* [109], *S. swartzii* [71], and *S. binderi* [74] have also demonstrated potential to inhibit *M. phaseolina* growth. Among brown algae, relevant inhibitory activity was also revealed by *Cystoseira indica* [109], *Dictyota indica*, *Padina tetrastomatica*, *S. polypodioides* (previously identified as *S. marginatum*) [71], *Stokeyia indica,* and *Spatoglossum variabile* [72,74]. As reported above for *Fusarium*, the brown algae extracts seem to be more effective than the remaining algae groups, which may be due to the presence of polyphenols [52] and/or 1-aminocyclopropane-1-carboxylic acid [71,113], which may also be the reason for their activity against *M. phaseolina*. The effectiveness of the dry powder obtained from *Melanothamnus afaqhusainii* [72,74] and *S. robusta* [71] demonstrated the potential of red algae in planta assays. The potential of the Rhodophyta group was also confirmed in vitro, namely, with *Centroceras* sp., *Ceramium* sp., *Gelidium pulchrumi*, *Gracilaria corticate*, *Halymenia porphyriformis*, *Hypnea musciformis*, *Jania pedunculata* var. *adhaerens*, *Neoporphyra perforate*, and *Osmundea pinnatifida* [52], which presented antifungal activity against *M. phaseolina*. Though to a lesser extent, the antifungal activity of green algae against this fungus species was also demonstrated in vitro with *C. racemosa*, *C. taxifolia*, *Chaetomorpha antennina*, *Codium indicum*, *Udotea* sp., and *Ulva rigida* [52], and also in planta using dry powder *Rhizoclonium implexum* and *H. tuna* [71,74]. Some of the compounds associated with the antifungal activity from macroalgae extracts are the volatile compounds in the essential oils [72], namely alcohols, aldehydes, carboxylic acids, ketones, esters, and hydrocarbons [114].

Similar to the studies performed with *Fusarium* species and *M. phaseolina*, Khan [52] also tested a diverse set of algae extracts against the growth of the soil-borne fungus *Rhizoctonia solani* [52]. Susceptibility to red, green, and brown algae was observed, but to a lesser extent than when the extracts were obtained using water instead of methanol [52]. Curiously, for some of the macroalgae, inhibitory activity was observed only with the methanolic extracts. The suppression of this fungus was influenced by the different compounds, which resulted from the use of different solvents during the macroalgae extraction procedure, highlighting the type of extraction as a major factor in obtaining antifungal compounds, with the methanolic extracts presenting an overall higher activity [52]. In the same study, a predominance of brown algae exhibiting antifungal activity (Table 2) was observed. This is in agreement with the high diversity of classes of compounds typically found in brown algae, confirming their compositional diversity and revealing their antifungal bioactivities [52]. This capacity is usually associated with phenolic compounds, specifically phlorotannins, which are very abundant in Phaeophyceae algae, and also with crinitol, an acyclic diterpene alcohol already described with antimicrobial activity against a wide range of microorganisms [115,116]. Recently, the chemical characterization by gas chromatography coupled to mass spectroscopy (GC–MS), of a brown alga extract, *Sargassum tenerrimum*, possessing inhibitory activity against spore germination/growth of *R. solani*, demonstrated a high abundance of n-hexadecanoic acid [79]. Considering the vestigial quantities of the remaining compounds analysed, the antifungal activity presented by *S. tenerrimum* was associated with this saturated long-chain fatty acid. However, this does not remove the need for more compositional tests with the remaining algae that also presented activity [79], as well as the isolation and analysis of specific compounds, to unravel the molecular mechanism underlying the antifungal activity of macroalgae extracts. Promising results were also obtained in planta with the crude algae and extracts obtained from a diverse group of green, red, and brown seaweeds against *R. solani* infection of soybean and pepper plants [71], eggplant, watermelon [72], cotton crops [74], sunflower, and tomato plants [75].

**Table 2:** Compilation of the best antifungal activities of macroalgae extracts against phytopathogenic fungi obtained using in vitro methodologies (summary of the information available in Scopus up until 25 February 2021). Detailed information regarding the results obtained for each study can be consulted in Supplementary Tables S1–S10. * Algae species not found in the Algaebase database.

Phytopathogenic Fungi	Host Species	Methodology	Reference
*Alternaria alternata*	*Hormophysa cuneiformis*	Agar diffusion assay/Broth microdilution assay	[56]
	*Ulva lactuca*	Disc diffusion technique	[58]
*Aspergillus fumigatus*	*Anthophycus longifolius*	Well diffusion technique	[51]
	*Osmundea pinnatifida*	Radial growth inhibition	[117]
*Aspergillus niger*	*Anthophycus longifolius*	Well diffusion technique	[51]
	*Ulva lactuca*	Disc diffusion technique	[58]
*Aspergillus terreus*	*Anthophycus longifolius*	Well diffusion technique	[51]
*Botrytis cinerea*	*Dictyopteris polypodioides*	Agar diffusion technique	[63]
*Cladosporium herbarum*	*Hormophysa cuneiformis*	Agar diffusion assay/Broth microdilution assay	[56]
*Colletotrichum acutatum*	*Dictyota dichotoma*	Disc diffusion technique	[65]
	*Dictyota implexa*	Disc diffusion technique	[65]
	*Dictyota spiralis*	Disc diffusion technique	[65]
*Colletotrichum falcatum*	*Caulerpa racemosa*	Poisoned food technique	[66]
	*Hydropuntia edulis*	Poisoned food technique	[66]
	*Sargassum myricocystum* *	Poisoned food technique	[66]
*Colletotrichum gloeosporioides*	*Hypnea musciformis*	Disc diffusion technique	[67,68]
	*Kappaphycus alvarezii*	Poisoned food technique	[69]
	*Laurencia dendroidea*	Disc diffusion technique	[67]
	*Ochtodes secundiramea*	Disc diffusion technique	[67,68]
	*Palisada flagellifera*	Disc diffusion technique	[68]
	*Pterocladiella capillacea*	Disc diffusion technique	[67]
*Colletotrichum musae*	*Hypnea musciformis*	Poisoned food technique	[67]
	*Laurencia dendroidea*	Poisoned food technique	[67]
	*Ochtodes secundiramea*	Poisoned food technique	[67]
	*Padina gymnospora*	Poisoned food technique	[67]
	*Pterocladiella capillacea*	Poisoned food technique	[67]
*Fusarium culmorum*	*Fucus vesiculosus*	Inhibition of mycelial growth/Macroconidia germination inhibition	[57]
*Fusarium graminearum*	*Dictyopteris polypodioides*	Agar diffusion technique	[63]
*Fusarium moniliforme*	*Botryocladia leptopoda*	Test tube in agar	[96]
	*Dictyota hauckiana*	Test tube in agar	[96]
*Fusarium oxysporum*	*Asparagopsis taxiformis*	Well diffusion technique	[118]
	*Calliblepharis floresii* *	Poisoned food technique	[52]
	*Caulerpa chemnitzia*	Poisoned food technique	[52]
	*Caulerpa racemosa*	Poisoned food technique	[52]
	*Caulerpa scalpelliformis*	Poisoned food technique	[52]
	*Caulerpa taxifolia*	Poisoned food technique	[52]
	*Centroceras* sp.	Poisoned food technique	[52]
	*Ceramium* sp.	Poisoned food technique	[52]
	*Chaetomorpha antennina*	Poisoned food technique	[52]
	*Codium indicum*	Poisoned food technique	[52]
	*Dictyopteris polypodioides*	Agar diffusion technique	[63]
	*Dictyota dicotoma*	Poisoned food technique	[52]
	*Gelidium pulchrum*	Poisoned food technique	[52]
	*Gracilaria corticata*	Poisoned food technique	[52]
	*Halimeda tuna*	Poisoned food technique/Field studies	[52,71]
	*Halymenia porphyriformis*	Poisoned food technique	[52]
	*Hormophysa cuneiformis*	Agar diffusion assay/Broth microdilution assay	[56]
	*Hypnea musciformis*	Poisoned food technique	[52]
	*Jania pedunculata* var. *adhaerens*	Poisoned food technique	[52]
	*Jolyna laminariodes*	Poisoned food technique	[52]
	*Melanothamnus afaqhusainii*	Poisoned food technique/Field studies	[52,72]
	*Neoporphyra perforata*	Poisoned food technique	[52]
	*Osmundea pinnatifida*	Poisoned food technique	[52]
	*Padina boergesenii*	Disc diffusion technique	[119]
	*Padina tetrastromatica*	Poisoned food technique	[52,71]
	*Polycladia indica*	Poisoned food technique/Disc diffusion technique	[52,71,72,109]
	*Polycladia myrica*	Disc diffusion technique	[119]
	*Sargassum aquifolium*	Poisoned food technique	[52]
	*Sargassum cinereum*	Disc diffusion technique	[119]
	*Sargassum ilicifolium*	Disc diffusion technique	[109]
	*Sargassum tenerrimum*	Poisoned food technique	[52]
	*Sargassum wightii*	Poisoned food technique	[52]
	*Scinaia huismanii*	Poisoned food technique	[52]
	*Spatoglossum asperum*	Disc diffusion assay	[120]
	*Steochospermum polypolides* *	Poisoned food technique	[52]
	*Udotea* sp.	Poisoned food technique	[52]
	*Ulva rigida*	Poisoned food technique	[52]
	*Valaniopsis* sp. *	Poisoned food technique	[52]
*Fusarium oxysporum albedinis*	*Dictyota dichotoma*	Disc diffusion technique	[65]
	*Dictyota implexa*	Disc diffusion technique	[65]
	*Dictyota spiralis*	Disc diffusion technique	[65]
*Fusarium oxysporum dianthi*	*Dictyota dichotoma*	Disc diffusion technique	[65]
	*Dictyota implexa*	Disc diffusion technique	[65]
	*Dictyota spiralis*	Disc diffusion technique	[65]
*Fusarium oxysporum* f.sp. *udum*	*Caulerpa racemosa*	Poisoned food technique	[105]
	*Hydropuntia edulis*	Poisoned food technique	[105]
	*Sargassum myricocystum* *	Poisoned food technique	[105]
*Fusarium oxysporum lycopersici*	*Dictyota dichotoma*	Disc diffusion technique	[65]
	*Dictyota implexa*	Disc diffusion technique	[65]
	*Dictyota spiralis*	Disc diffusion technique	[65]
*Fusarium solani*	*Botryocladia leptopoda*	Test tube in agar	[96]
	*Caulerpa racemosa*	Test tube in agar	[96]
	*Caulerpa taxifolia*	Test tube in agar	[96]
	*Champia compressa*	Test tube in agar	[96]
	*Codium indicum*	Test tube in agar	[96]
	*Gracilaria corticata*	Test tube in agar	[96]
	*Hypnea musciformis*	Test tube in agar	[96]
	*Hypnea valentiae*	Test tube in agar	[96]
	*Osmundea pinnatifida*	Test tube in agar	[96]
	*Padina antillarum*	Test tube in agar	[96]
	*Sarconema filiforme*	Test tube in agar	[96]
	*Sargassum ilicifolium*	Test tube in agar	[96]
	*Sargassum vulgare*	Test tube in agar	[96,121]
	*Solieria robusta*	Test tube in agar/Field studies	[71,74,96,121]
	*Spatoglossum asperum*	Disc diffusion assay	[120]
	*Stoechospermum polypodioides*	Test tube in agar/Field studies	[71,74,96]
	*Ulva lactuca*	Test tube in agar	[96]
*Fusarium* sp.	*Anthophycus longifolius*	Well diffusion technique	[51]
*Ganoderma boninense*	*Caulerpa lamourouxii*	Poisoned food technique	[122]
	*Caulerpa racemosa*	Poisoned food technique	[122]
	*Halimeda macrophysa*	Poisoned food technique	[122]
	*Sargassum oligocystum*	Poisoned food technique	[122]
*Geotrichum* sp.	*Dictyopteris polypodioides*	Agar diffusion technique	[63]
*Macrophomina phaseolina*	*Calliblepharis floresii* *	Poisoned food technique	[52]
	*Caulerpa racemosa*	Poisoned food technique	[52]
	*Caulerpa taxifolia*	Poisoned food technique	[52]
	*Centroceras* sp.	Poisoned food technique	[52]
	*Ceramium* sp.	Poisoned food technique	[52]
	*Chaetomorpha antennina*	Poisoned food technique	[52]
	*Codium indicum*	Poisoned food technique	[52]
	*Dictyota dicotoma*	Poisoned food technique	[52]
	*Gelidium pulchrum*	Poisoned food technique	[52]
	*Gracilaria corticata*	Poisoned food technique	[52]
	*Halymenia porphyriformis*	Poisoned food technique	[52]
	*Hypnea musciformis*	Poisoned food technique	[52]
	*Jania pedunculata* var. *adhaerens*	Poisoned food technique	[52]
	*Jolyna laminariodes*	Poisoned food technique	[52]
	*Melanothamnus afaqhusainii*	Poisoned food technique	[52]
	*Neoporphyra perforata*	Poisoned food technique	[52]
	*Osmundea pinnatifida*	Poisoned food technique	[52]
	*Padina tetrastromatica*	Poisoned food technique	[52]
*Macrophomina phaseolina*	*Polycladia indica*	Poisoned food technique/Disc diffusion technique	[52,109]
	*Sargassum aquifolium*	Poisoned food technique	[52]
	*Sargassum ilicifolium*	Disc diffusion technique	[109]
	*Sargassum tenerrimum*	Poisoned food technique	[52]
	*Sargassum wightii*	Poisoned food technique	[52]
	*Scinaia huismanii*	Poisoned food technique	[52]
	*Spatoglossum asperum*	Disc diffusion assay	[120]
	*Stoechospermum polypodioides*	Poisoned food technique	[52]
	*Udotea* sp.	Poisoned food technique	[52]
	*Ulva rigida*	Poisoned food technique	[52]
	*Valaniopsis* sp. *	Poisoned food technique	[52]
*Mucor* sp.	*Champia compressa*	Test tube in agar	[96]
	*Hypnea musciformis*	Test tube in agar	[96]
	*Sargassum boveanum*	Test tube in agar	[96]
	*Sargassum ilicifolium*	Test tube in agar	[96]
	*Ulva lactuca*	Test tube in agar	[96]
*Penicillium expansum*	*Ulva lactuca*	Disc diffusion technique	[58]
*Penicillium* sp.	*Dictyota dichotoma*	Disc diffusion technique	[123]
	*Ulva lactuca*	Disc diffusion technique	[123]
*Penicillum digitatum*	*Hormophysa cuneiformis*	Agar diffusion assay/Broth microdilution assay	[56]
*Phialophora cinerescens*	*Dictyota dichotoma*	Disc diffusion technique	[65]
	*Dictyota implexa*	Disc diffusion technique	[65]
	*Dictyota spiralis*	Disc diffusion technique	[65]
*Phoma tracheiphila*	*Dictyota dichotoma*	Disc diffusion technique	[65]
	*Dictyota implexa*	Disc diffusion technique	[65]
	*Dictyota spiralis*	Disc diffusion technique	[65]
*Pseudocercospora fijiensis*	*Halymenia floresii*	Minimum inhibitory concentration	[94]
*Pyricularia oryzae*	*Rhodomela confervoides*	Spore spreading method	[95]
	*Symphyocladia latiuscula*	Spore spreading method	[95]
*Rhizoctonia solani*	*Calliblepharis floresii*	Poisoned food technique	[52]
	*Centroceras* sp.	Poisoned food technique	[52]
	*Ceramium* sp.	Poisoned food technique	[52]
	*Chaetomorpha antennina*	Poisoned food technique	[52]
	*Codium indicum*	Poisoned food technique	[52]
	*Dictyopteris undulata*	Fungitoxic activity	[92]
	*Gelidium pulchrum*	Poisoned food technique	[52]
	*Gracilaria corticata*	Poisoned food technique	[52]
	*Halymenia porphyriformis*	Poisoned food technique	[52]
	*Hypnea musciformis*	Poisoned food technique	[52]
	*Jania pedunculata* var. *adhaerens*	Poisoned food technique	[52]
	*Melanothamnus afaqhusainii*	Poisoned food technique	[52]
	*Neoporphyra perforata*	Poisoned food technique	[52]
	*Osmundea pinnatifida*	Poisoned food technique	[52]
	*Padina tetrastromatica*	Poisoned food technique	[52]
	*Polycladia indica*	Poisoned food technique	[52]
	*Sargassum aquifolium*	Poisoned food technique	[52,71,74]
	*Sargassum tenerrimum*	Poisoned food technique	[52,71]
*Rhizoctonia solani*	*Sargassum wightii*	Poisoned food technique	[52]
	*Spatoglossum asperum*	Disc diffusion assay/Field studies	[73,120]
	*Stoechospermum polypodioides*	Poisoned food technique/Field studies	[52,71,74]
	*Udotea* sp.	Poisoned food technique	[52]
	*Ulva rigida*	Poisoned food technique	[52]
	*Valaniopsis* sp. *	Poisoned food technique	[52]
	*Dictyota dichotoma*	Disc diffusion technique/Spore germination	[79]
	*Padina gymnospora*	Disc diffusion technique/Spore germination	[79]
	*Sargassum muticum*	Disc diffusion technique/Spore germination	[79]
	*Sargassum tenerrimum*	Disc diffusion technique/Spore germination	[79]
	*Sargassum wightii*	Disc diffusion technique/Spore germination	[79]
*Sclerotinia sclerotiorum*	*Dictyopteris undulata*	Fungitoxic activity	[92]
*Sclerotium rolfsii*	*Dictyopteris undulata*	Fungitoxic activity	[92]
*Verticillium dahliae*	*Cystoseira humilis* var. *myriophylloides*	Poisoned food technique	[93]
	*Dictyopteris polypodioides*	Agar diffusion technique	[63]
	*Fucus spiralis*	Poisoned food technique	[93]

#### 3.2.2. Potential Antifungal Mechanisms

The mode of action of antifungal compounds extracted by macroalgae is still poorly understood. Generally, a fungus can be affected by compounds directly targeting the cell wall or membrane, two important components that contact with the exterior environment, or intracellular organelles, such as nucleic acids or mitochondria. Antifungal agents that enter into the cell can disrupt protein synthesis by their interaction with nucleic acids [124], as well as disturb the homeostasis and stability of the cell by interfering with the mitochondrial respiratory chain [125,126].

An important target usually affected by commercial antifungal products is the fungal membrane [124,126]. The cell membrane is a primary and crucial component for guaranteeing cellular stability in a fungal organism [53]. Abnormalities and events occurring at the membrane level can disturb cell stability, leading to the reduction of cell lifespan [127]. Fatty acids are a vast and diversified group of compounds present in macroalgae and have been mentioned several times throughout this work due to their antifungal potential. The unique composition of fatty acids, characterized by the presence of a carboxyl group at one end and a methyl group at the other chain end, allows their insertion into the fungal membrane, promoting an increase of fluidity and, consequently, their permeability, modifying their conformational organization and culminating in cell death [54]. This antifungal mechanism was demonstrated by Hajlaou and colleagues against relevant fungal species, such as *Cladosporium cucumerinum*, *B. cinerea* and *Fusarium oxysporum* f.sp. *radicislycopersici* [128], affecting conidia germination and fungal biomass production.

**Figure 1:**
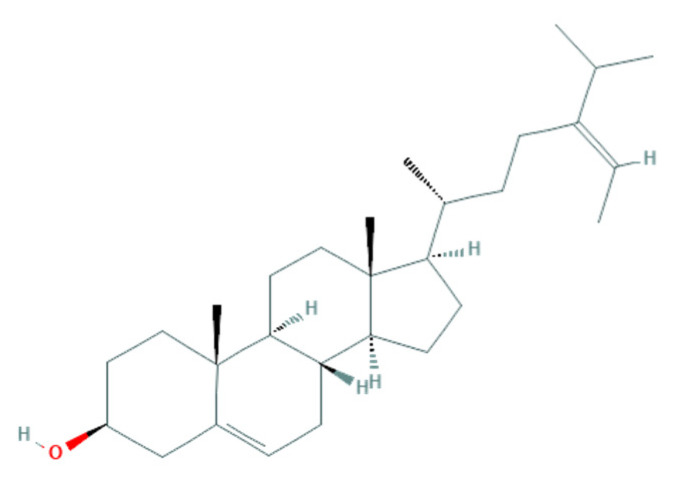
Fucosterol (C_29_H_48_O). Chemical structure obtained from PubChem database on 4 August 2021 (https://pubchem.ncbi.nlm.nih.gov/compound/5281328#section=2D-structure).

**Figure 2:**
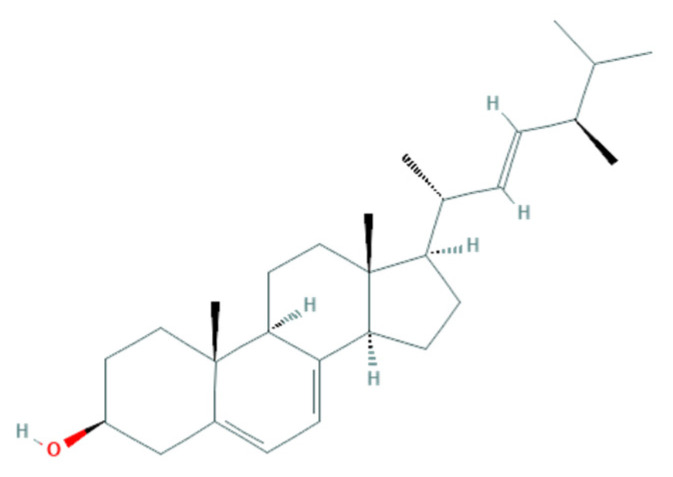
Ergosterol (C_28_H_44_O). Chemical structure obtained from PubChem database 4 August 2021 (https://pubchem.ncbi.nlm.nih.gov/compound/444679#section=2D-structure).

**Figure 3:**
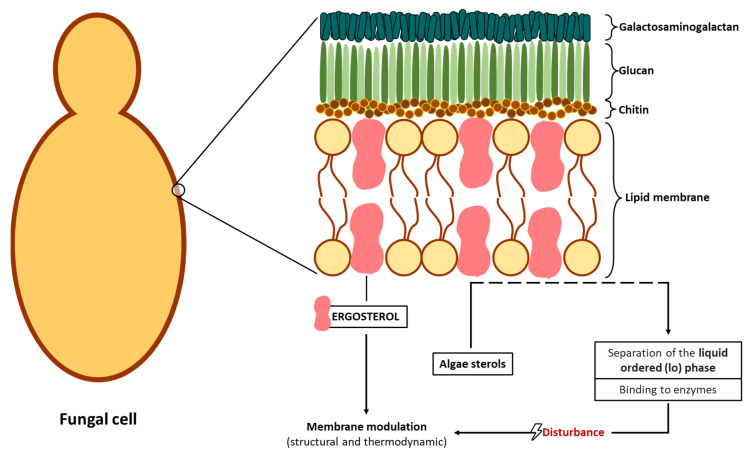
Schematic representation of a possible interaction of algae sterols in the fungal membrane.

Another antifungal mechanism proposed is related to sterol present in the fungal membrane. Some algae compounds have the capacity to interact/inhibit sterol synthesis [124]. One example is observed with the algae-based products of *F. vesiculosus*, presenting a high content of fucosterol, a natural sterol isolated from brown algae, known to possess fungistatic and antifungal activity against *F. culmorum* [57]. The similarity of this algae-derived sterol (Figure 1) with ergosterol (Figure 2) (a sterol in the fungal membrane, responsible for stability) allows the interaction of fucosterol with fungal membrane modulators to disturb their normal regulation (Figure 3) [129], as well as the increase of the fluidity of the membrane components [53].

Another antifungal mechanism is demonstrated by *Candida* spp. [130] against filamentous phytopathogenic fungi. This action is related to the chemical characterization of unsaturated fatty acids (defined by one or more C=C bond/s), which can improve the antifungal action of these compounds. This property is associated with the easy incorporation of polyunsaturated lipids into the fungal membrane, which also contributes to the destabilization of cell structure, triggering events of oxidative stress [128] known to act against several species of phytopathogenic fungi, such as *Alternaria solani*, *A. niger*, *B. cinerea*, *C. cucumerinum*, *F. oxysporum*, and *Rh. solani* [53].

Another important group of compounds presenting antifungal potential are phenolic compounds. Among them, phlorotannins are highlighted as one of the relevant antifungal compounds of brown algae, as presented above. However, the antifungal mechanism of these compounds has only been clarified for yeast species [126,131].

## 4. In Planta Studies: Are These Assays Enough to Prove the Antifungal Potential of the Extracts?

The assays performed in vivo, in this case with the use of algae extracts on the host plant, are a peculiar case of a complex analysis, more difficult than in vitro assays. The suppression of infection/colonies in the host tissues can be a consequence of two possible situations: (1) a direct antifungal action over the phytopathogenic agent, or (2) an elicitation, promoting the activation of defense pathways of the plant.

Table 3 presents the most relevant assays performed in field/greenhouse conditions against fungal phytopathogenic species. Several studies have demonstrated the antifungal potential of dry powder macroalgae in field/greenhouse conditions against several phytopathogenic fungi, such as *Fusarium* species. Ehteshamul-Haque and colleagues [71] tested the inhibition potential of the brown algae *Dictyota cervicornis* (identified as *Dictyota indica*), *Padina tetrastromatica*, *Stoechospermum polypodioides* (then identified as *Stoechospermum marginatum*), *Polycladia indica* (as *Stokeyia indica*), *Sargassum swartzii*, the red alga *Solieria robusta,* and the green alga *Halimeda tuna* against the root-rotting fungi *Fusarium* spp., in *Glycine max* Merrill and *Capsicum annuum* plants [71]. 1-Aminocyclopropane-1-carboxylic acid (ACC) was suggested to be responsible for the antimicrobial activity displayed by the algae [71,72,75], but the lack of an in vitro test hampers this conclusion. Thus, it is of major importance to combine both in vivo and in vitro tests in order to better understand the interaction between the extract, fungal phytopathogen, and host.

A similar situation to the one described in the first paragraph of this section was observed in another in planta assay. Despite the infection inhibition/suppression success obtained against *M. phaseolina* [52,71,72,74] and *R. solani* [71,72,74,75] after the application of a dry powder from a diverse group of green, red, and brown algae in plants such as soybean, pepper, eggplant, watermelon, cotton crops, sunflower, and tomato (greenhouse and/or field conditions), a direct antifungal activity cannot be attributed to the macroalgae based only on these assays. Additionally, in vitro tests have been performed with ethanolic extracts of some common macroalgae, as referred to in Table 2, Section 3, namely, *H. tuna* against *M. phaseolina* and *R. solani*, and *Sargassum swartzii* [71] and *Melanothamnous afaqhusainii* [72,74,75] against *R. solani* [109]. By using the disc diffusion method, no activity was noticed against these fungi [109], but in planta tests of the same macroalgae dry powder found that it inhibited the infection caused by these phytopathogens [71,74]. This could mean that either the antifungal compounds do not belong to the ethanolic fraction, which is unexpected, as the extracted lipophilic compounds are the ones reported to possess antifungal activity [66,132], or a direct antifungal activity is not the cause of infection suppression. The latter possibility seems plausible since dried macroalgae are also known to stimulate the growth of plants, as well as to contribute to a higher resistance against microorganisms, through the activation of intrinsic defence pathways [133,134,135,136]. This way, in the context of fighting fungal infections, one should include the potential of the compounds to act as elicitors, promoting the defence mechanisms of the plants, instead of direct antifungal activity against the phytopathogenic fungi, which, of course, triggers the need for different assessment strategies for algae extracts.

## 5. Conclusions

This review gives a résumé of all available information concerning the antifungal activity of macroalgae extracts against phytopathogenic fungi. A strong inhibitory capacity is ubiquitous among all different macroalgae groups, but the potential of brown algae is predominant. Fatty acids, phenolic compounds, terpenoids and their derivatives, and polysaccharides are some of the compounds of macroalgal origin responsible for inhibitory activity against the phytopathogenic fungi. Notwithstanding the number of available works in the area, more efforts are still needed to elucidate the specific compounds responsible for antifungal action, their chemical structures, and the mechanisms of action.

The enormous potential of a natural source of antifungal compounds is frequently seen as the future to combat the “silent fungal crisis” spread all over the world. The effectiveness of macroalgae-derived compounds is yet not fully disclosed and their potential introduction for agricultural purposes may reveal the onset of eco-friendly strategies, not only as antifungal agents, but also as elicitors of plant defence pathways.

Despite the natural sourcing, which gives increased societal acceptability, the optimization of assays that allow understanding of the influence of macroalgae compounds in non-target species is paramount to achieve the twofold goal of efficiency and low environmental impact. More studies conducted in field are necessary to ensure that the ability to control the development of fungal plant pathogens are not only present in in vitro tests but also in real conditions. The biotechnological use of marine resources for agriculture is still in its infancy, but the increased number of studies pinpointing their potential and success promises a future where the use of these natural compounds may further contribute to scaling up food supply and enhancing food security in order to meet the increasing demands for quality products from an ever-increasing population.

## Figures and Tables

**Table 3:** Compilation of the best results obtained by macroalgae extracts/dry powder against plants infected with phytopathogenic fungi using in field methodologies (summary of the information available in Scopus up until 25 February 2021). * Infection expressed in % of infection after application of the macroalgae extract. A: The antimicrobial activity can be due the presence of acrylic acid; B: Increase in effectiveness after a second application; C: Loss of effectiveness for long periods.

Phytopathogenic Fungi	Macroalgae Source	Extract Type/Concentration	In field Assays		Greenhouse Assays Green		References
			Host	Infection *	Host	Infection *	
*Fusarium oxysporum*	*Dictyota cervicornis*	Dry powder	*Glycine max* (L.)	6.2	-	-	[71]
	*Halimeda tuna*	Dry powder	*Glycine max* (L.)	0	-	-	[71]
	*Melanothamnus afaqhusainii*	Dry powder	Eggplant	0	-	-	[72]
		Dry powder	Watermelon	0	-	-	[72]
	*Padina tetrastromatica*	Dry powder	*Glycine max* (L.)	0	-	-	[71]
	*Polycladia indica*	Dry powder	Eggplant	0	-	-	[72]
		Dry powder	Watermelon	0	-	-	[72]
	*Sargassum swartzii*	Dry powder	*Glycine max* (L.)	12.5	-	-	[71]
	*Solieria robusta*	Dry powder	*Glycine max* (L.)	0	-	-	[71]
	*Spatoglossum variabile*	Dry powder	Eggplant	0	-	-	[72]
	*Stoechospermum polypodioides*	Dry powder	*Glycine max* (L.)	0	-	-	[71]
*Fusarium solani*	*Dictyota cervicornis*	Dry powder	*Glycine max* (L.)	0	*Glycine max* (L.)	6.2	[71]
	*Halimeda tuna*	Dry powder	*Gossypium hirsutum* L.	0	*Gossypium hirsutum* L.	12.5	[74]
		Dry powder	Sunflower	0	*Glycine max* (L.)	12.5	[71]
		Dry powder	*-*	-	*Lycopersicum esculentum*	12.5 ^A^	[75]
	*Melanothamnus afaqhusainii*	Dry powder	*Lycopersicum esculentum*	0 ^A^	Sunflower	25 ^A^	[75]
	*Padina tetrastromatica*	Dry powder	*Glycine max* (L.)	0	*Glycine max* (L.)	12.5	[71]
		Dry powder	*Capsicum annum* L.	0	*-*	-	[71]
	*Polycladia indica*	Dry powder	*Capsicum annum* L.	6.2	*Glycine max* (L.)	18.7	[71]
		Dry powder	*Gossypium hirsutum* L.	12.5	*Gossypium hirsutum* L.	6.2	[74]
	*Rhizoclonium riparium*	Dry powder	*Gossypium hirsutum* L.	18.7 ^C^	*Gossypium hirsutum* L.	18.7	[74]
	*Sargassum aquifolium*	Dry powder	*Capsicum annum* L.	6.2	*Gossypium hirsutum* L.	6.2	[71,74]
	*Sargassum swartzii*	Dry powder (0.5%)	*-*	-	*Lycopersicum esculentum*	31.2	[73]
		Dry powder (1%)	*-*	-	*Lycopersicum esculentum*	12.5	[73]
		Dry powder	*Glycine max* (L.)	0 ^C^	*Glycine max* (L.)	6.2	[71]
	*Sargassum tenerrimum*	Dry powder	*Capsicum annum* L.	6.2 ^B^	-	-	[71]
	*Solieria robusta*	Dry powder	*Glycine max* (L.)	6.2 ^C^	*Glycine max* (L.)	0	[71]
		Dry powder	*Capsicum annuum* L.	0 ^B^	*Gossypium hirsutum* L.	12.5	[71,74]
	*Spatoglossum asperum*	Dry powder (0.5%)	*Solanum melongena* L.	18.7	*-*	-	[72]
	*Spatoglossum variabile*	Dry powder	*Citrullus lanatus* (Thunb.) Matsum. & Nakai	18.7	*Gossypium hirsutum* L.	18.7	[72,74]
		Dry powder	Sunflower	0 ^A^	Sunflower	18.7 ^A^	[75]
		Dry powder	*Lycopersicum esculentum*	0 ^A^	*Lycopersicum esculentum*	12.5 ^A^	[75]
	*Stoechospermum polypodioides*	Dry powder	*Glycine max* (L.)	0 ^C^	*Glycine max* (L.)	12.5	[71]
		Dry powder	*Capsicum annuum* L.	6.2	*Gossypium hirsutum* L.	18.7	[71,75]
*Macrophomina phaseolina*	*Dictyota cervicornis*	Dry powder	*Glycine max* (L.)	6.2	*Glycine max* (L.)	6.2	[71]
	*Halimeda tuna*	Dry powder	*Glycine max* (L.)	0	*Glycine max* (L.)	0	[71]
		Dry powder	*Capsicum annuum* L.	0 ^C^	Sunflower	12.5 ^A^	[75]
		Dry powder	*Gossypium hirsutum* L.	0 ^A^	*Gossypium hirsutum* L.	18.7	[74,75]
	*Melanothamnus afaqhusainii*	Dry powder	*Solanum melongena* L.	12.5	Sunflower	18.7 ^A^	[72,75]
			*Citrullus lanatus* (Thunb.) Matsum. & Nakai	0	*-*	-	[72]
		Dry powder	*Gossypium hirsutum* L.	6.2	*Gossypium hirsutum* L.	12.5	[74]
		Dry powder	*Lycopersicum esculentum*	0 ^A^	*Lycopersicum esculentum*	0 ^A^	[75]
	*Padina tetrastromatica*	Dry powder	*Capsicum annuum* L.	0	-	-	[71]
	*Polycladia indica*	Dry powder	*Glycine max* (L.)	12.5	*Glycine max* (L.)	0	[71]
		Dry powder	*Capsicum annuum* L.	0 ^C^	-	-	[71]
		Dry powder	*Gossypium hirsutum* L.	6.2	*Gossypium hirsutum* L.	25	[74]
		Dry powder	*Solanum melongena* L.	0	*-*	-	[72]
		Dry powder	*Citrullus lanatus* (Thunb.) Matsum. & Nakai	0	*-*	-	[72]
	*Rhizoclonium riparium*	Dry powder	*Gossypium hirsutum* L.	12.5	*Gossypium hirsutum* L.	6.2	[74]
	*Sargassum aquifolium*	Dry powder	*Capsicum annuum* L.	0	-	-	[71]
		Dry powder	*Gossypium hirsutum* L.	12.5	*Gossypium hirsutum* L.	12.5	[74]
	*Sargassum swartzii*	Dry powder (0.5%)	-	-	*Lycopersicum esculentum*	0	[73]
		Dry powder (1%)	-	-	*Lycopersicum esculentum*	0	[73]
	*Sargassum tenerrimum*	Dry powder	*Capsicum annuum* L.	0 ^C^	-	-	[71]
	*Solieria robusta*	Dry powder	*Glycine max* (L.)	0	*Glycine max* (L.)	0	[71]
		Dry powder	*Gossypium hirsutum* L.	0	*Gossypium hirsutum* L.	18.7	[74]
		Dry powder	*Capsicum annuum* L.	0	*-*	-	[71]
	*Spatoglossum asperum*	Dry powder (0.5%)	-	-	*Lycopersicum esculentum*	6.2	[73]
		Dry powder (1%)	-	-	*Lycopersicum esculentum*	0	[73]
	*Spatoglossum variabile*	Dry powder	Sunflower	0 ^A^	Sunflower	0 ^A^	[75]
		Dry powder	*Lycopersicum esculentum*	0	*Lycopersicum esculentum*	0	[75]
		Dry powder	*Gossypium hirsutum* L.	6.2	*Gossypium hirsutum* L.	6.2	[74]
		Dry powder	*Solanum melongena* L.	0	*-*	-	
		Dry powder	*Citrullus lanatus* (Thunb.) Matsum. & Nakai	0	*-*	-	[72]
	*Stoechospermum polypodioides*	Dry powder	*Glycine max* (L.)	0 ^C^	*Glycine max* (L.)	6.2	[71]
			*Gossypium hirsutum* L.	0	*Gossypium hirsutum* L.	12.5	[74]
		Dry powder	*Capsicum annuum* L.	0	-	-	[71]
*Rhizoctonia solani*	*Dictyota cervicornis*	Dry powder	*Glycine max* L.	6.2 ^C^	*Glycine max* L.	0	[71]
	*Halimeda tuna*	Dry powder	*Capsicum annuum* L.	0 ^B^	*Glycine max* L.	0	[71]
			Sunflower	0 ^A^	*Gossypium hirsutum* L.	12.5	[74,75]
		Dry powder	*Lycopersicum esculentum*	12.5 ^A^	*Lycopersicum esculentum*	6.2 ^A^	[75]
	*Melanothamnus afaqhusainii*	Dry powder	*Citrullus lanatus*	0	*Gossypium hirsutum* L.	18.7	[72,74]
		Dry powder	*Lycopersicum esculentum*	12.5 ^A^	*Lycopersicum esculentum*	6.2 ^A^	[75]
		Dry powder	*-*	-	Sunflower	18.7	[75]
	*Padina tetrastromatica*	Dry powder	*Glycine max* L.	12.5 ^C^	*Glycine max* L.	0	[71]
		Dry powder	*Capsicum annuum* L.	0	-	-	[71]
	*Polycladia indica*	Dry powder	*Solanum melongena* L.	0	-	-	[71,72,74]
		Dry powder	*Citrullus lanatus* (Thunb.) Matsum. & Nakai	12.5	-	-	[72]
		Dry powder	*Capsicum annuum* L.	0	*Glycine max* L.	0	[71]
		Dry powder	*Gossypium hirsutum* L.	6.2	*Gossypium hirsutum* L.	12.5	[74]
	*Rhizoclonium riparium*	Dry powder	*Gossypium hirsutum* L.	25	*Gossypium hirsutum* L.	25	[74]
	*Sargassum aquifolium*	Dry powder	*Capsicum annuum* L.	0	*Gossypium hirsutum* L.	6.2	[71,74]
		Dry powder	*Gossypium hirsutum* L.	18.7	-	-	[74]
	*Sargassum swartzii*	Dry powder (0.5%)	*-*	-	*Lycopersicum esculentum*	0	[71,73]
		Dry powder (1%)	*-*	-	*Lycopersicum esculentum*	0	[71,73]
		Dry powder	*-*	-	*Glycine max* L.	0	[71]
	*Sargassum tenerrimum*	Dry powder	*Capsicum annuum* L.	0 ^C^	-	-	[71]
	*Solieria robusta*	Dry powder	*Capsicum annuum* L.	0	*Glycine max* L.	0	[71]
		Dry powder	*Gossypium hirsutum* L.	0	*Gossypium hirsutum* L.	12.5	[74]
	*Spatoglossum asperum*	Dry powder (0.5%)	*-*	-	*Lycopersicum esculentum*	25	[73]
		Dry powder (1%)	*-*	-	*Lycopersicum esculentum*	6.2	[73]
	*Spatoglossum variabile*	Dry powder	*Citrullus lanatus* (Thunb.) Matsum. & Nakai	0	Sunflower	12.5 ^A^	[72,75]
		Dry powder	*Lycopersicum esculentum*	12.5 ^A^	*Lycopersicum esculentum*	6.2 ^A^	[75]
	*Stoechospermum polypodioides*	Dry powder	*Capsicum annuum* L.	0	*Glycine max* L.	0	[71]

## Data Availability

Not applicable.

## Supplementary Materials

The following are available online at https://www.mdpi.com/article/10.3390/jof7121006/s1, Table S1: Data available about the antifungal activity against phytopathogenic fungi from macroalgae using the disc/well diffusion technique; Table S2: Data available about the antifungal activity against phytopathogenic fungi from macroalgae using the modified diffusion technique; Table S3: Data available about the antifungal activity against phytopathogenic fungi from macroalgae using the poisoned food technique; Table S4: Data available about the antifungal activity against phytopathogenic fungi from macroalgae using the poisoned food technique (data expressed in mycelial growth); Table S5: Data available about the antifungal activity against phytopathogenic fungi from macroalgae by the evaluation of macroconidia germination; Table S6: Data available about the antifungal activity against phytopathogenic fungi from macroalgae using the broth microdilution assay; Table S7: Data available about the antifungal activity against phytopathogenic fungi from macroalgae by the evaluation of inhibition of mycelial growth (by spraying the fungi culture with macroalgae extract); Table S8: Data available about the antifungal activity against phytopathogenic fungi from macroalgae by the evaluation of fungal spore germination; Table S9: Data available about the antifungal activity against phytopathogenic fungi from macroalgae by the spore spreading method; Table S10: Data available about the antifungal activity against phytopathogenic fungi from macroalgae by the fungal germination in test tube; Table S11: Data available about the antifungal activity against phytopathogenic fungi from macroalgae tested in field studies; Table S12: Data available about the antifungal activity against phytopathogenic fungi from macroalgae tested in screenhouse studies.
