# Supplementary material for: Marine Macroalgae, a Source of Natural Inhibitors of Fungal Phytopathogens
Source: J Fungi (Basel). 2021 Nov 25;7(12):1006. doi: 10.3390/jof7121006 (PMC8708330; doi:10.3390/jof7121006)
Supplement: Supplementary file 1 [file jof-07-01006-s001.zip › jof-1444770-supplementary.pdf]

Table S1. Data available about the antifungal activity against phytopathogenic fungi from macroalgae using the disc/well diffusion technique.

Disc/Well Diffusion technique									
**Phytopathogenic fungi**	**Macroalgae source**	**Extraction solvent**	**Extract composition**	**Extract composition (bibliographic information)**	**Extract concentration /volume**	**Inhibition halo (mm)/ Growth inhibition (%)**	**Incubation conditions**	**Notes**	**Reference**
Aspergillus brasiliensis	*Saccharina japonica*	Supercritical fluid-CO_2 + Ethanol	High total fatty acids content + Elaidic acid, eicosapentaenoic acid, fucoxanthin	-	100 µg/mL (10 µL)	16 ± 0.00 mm	37ºC, 24-48h	-	[1]
		Acetone + methanol		-		20 ± 0.10 mm		-	
	Sargassum horneri	Supercritical fluid-CO_2 + Ethanol	High total fatty acids content + Palmitic acid, eicosapentaenoic acid, fucoxanthin	-		18 ± 0.50 mm		-	
		Acetone + methanol		-		28 ± 0.85 mm		-	
Aspergillus flavus	*Asparagopsis taxiformis*	Methanol	Chemical constituents of the main active fraction: 4,5-dimethyl-1H-pyrrole-2-carboxylic acid ethyl ester (56.012%), fatty acids, 14-methyl-pentadecanoic acid methyl ester (26.6%), octadecanoic acid methyl ester (8.46%), octadec-9-enoic acid 2,3-dihydroxy-propyl ester (4.11%), 9-octadecanoic acid, methyl ester (4.535%) and trace amount of chlorobenzene (0.09%).	-	10 mg/mL	14 ± 1.2 mm	30ºC, 72h	-	[2]
	Sargassum wightii	Acetone	Steroids, terpenoids, glycosides, alkaloids, flavonoids, tannins and saponins	-	-	9 ± 0.7 mm	-	-	[3]
		Methanol		-	-	12.6 ± 0.2 mm	-	-	
Aspergillus fumigatus	*Anthophycus longifolius*	Benzene	Proteins and amino acids, flavonoids, phenolic compounds, alkaloids, sugar/glucosides	-	Standard concentration	5.666 ± 0.353 mm*[A]	37ºC, 24h	-	[4]
		Acetic acid	Proteins and amino acids, glycosides, phenolic compounds, carbohydrates, alkaloids, sugar/glucosides.	-	Standard concentration	5.4 ± 0.231 mm*[A]	37ºC, 24h	-	
		Hexane	Proteins and amino acids, flavonoids, phenolic compounds, carbohydrates,	-	Standard concentration	8.166 ± 0.166 mm*[A]	37ºC, 24h	-	

Disc/Well Diffusion technique

			alkaloids, coumarine, sugar/glucosides.						
		DMSO	Proteins and amino acids, flavonoids, glycosides, phenolic compounds, alkaloids, sugar/glucosides.	-	Standard concentration	5.166 ± 0.088 mm*[A]	37°C, 24h	-	
		Diethyl ether	Proteins and amino acid, phenolic compounds, alkaloids, coumarine, sugar/glucosides.	-	Standard concentration	12.16 ± 0.088 mm*[A]	37°C, 24h	-	
		Chloroform	Proteins and amino acids, flavonoids, glycosides, phenolic compounds, alkaloids, coumarine, sugar/glucosides.	-	Standard concentration	9.166 ± 0.166 mm*[A]	37°C, 24h	-	
Aspergillus niger	*Anthophycus longifolius*	Benzene	Proteins and amino acids, flavonoids, phenolic compounds, alkaloids, sugar/glucosides	-	Standard concentration	10.23 ± 0.145 mm*[A]	37°C, 24h	-	[4]
		Acetic acid		-	Standard concentration	9.3 ± 0.208 mm*[A]	37°C, 24h	-	
		Hexane	Proteins and amino acids, flavonoids, phenolic compounds, carbohydrates, alkaloids, coumarine, sugar/glucosides.	-	Standard concentration	9.06 ± 0.066 mm*[A]	37°C, 24h	-	
		DMSO	Proteins and amino acids, flavonoids, glycosides, phenolic compounds, alkaloids, sugar/glucosides.	-	Standard concentration	14.4 ± 0.305 mm*[A]	37°C, 24h	-	
		Diethyl ether	Proteins and amino acid, phenolic compounds, alkaloids, coumarine, sugar/glucosides.	-	Standard concentration	14.06 ± 0.066 mm*[A]	37°C, 24h	-	
		Chloroform	Proteins and amino acids, flavonoids, glycosides, phenolic compounds, alkaloids, coumarine, sugar/glucosides.	-	Standard concentration	13.23 ± 0.145 mm*[A]	37°C, 24h	-	
	Asparagopsis taxiformis	Methanol	Chemical constituints of the main active fraction: 4,5-dimethyl-1H-pyrrole-2-carboxylic acid ethyl ester (56.012%), fatty acids, 14-methyl-pentadecanoic acid methyl ester (26.6%), octadecanoic acid methyl	-	10 mg/mL	13 ± 1.4 mm	30°C, 72h	-	[2]

Disc/Well Diffusion technique									
			ester (8.46%), octadec-9-enoic acid 2,3-dihydroxy-propyl ester (4.11%), 9-octadecanoic acid, methyl ester (4.535%) and trace amount of chlorobenzene (0.09%).						
	Sargassum wightii	Acetone	Steroids, terpenoids, glycosides, alkaloids, flavonoids, tannins and saponins	-	-	11.3 ± 0.4 mm	-	-	[3]
		Methanol	Steroids, terpenoids, alkaloids, flavonoids, tannins and saponins	-	-	14.6 ± 0.9 mm	-	-	[3]
Aspergillus terreus	*Anthophycus longifolius*	Benzene	Proteins and amino acids, flavonoids, phenolic compounds, alkaloids, sugar/glucosides	-	Standard concentration	5.833 ± 0.929 mm[*A]	37°C, 24h	-	[4]
		Acetic acid	Proteins and amino acids, glycosides, phenolic compounds, carbohydrates, alkaloids, sugar/glucosides.	-	Standard concentration	6.466 ± 0.240 mm[*A]	37°C, 24h	-	[4]
		Hexane	Proteins and amino acids, flavonoids, phenolic compounds, carbohydrates, alkaloids, coumarine, sugar/glucosides.	-	Standard concentration	9.23 ± 0.145 mm[*A]	37°C, 24h	-	[4]
		DMSO	Proteins and amino acids, flavonoids, glycosides, phenolic compounds, alkaloids, sugar/glucosides.	-	Standard concentration	9.73 ± 0.371 mm[*A]	37°C, 24h	-	[4]
		Diethyl ether	Proteins and amino acid, phenolic compounds, alkaloids, coumarine, sugar/glucosides.	-	Standard concentration	11.9 ± 0.379 mm[*A]	37°C, 24h	-	[4]
		Chloroform	Proteins and amino acids, flavonoids, glycosides, phenolic compounds, alkaloids, coumarine, sugar/glucosides.	-	Standard concentration	8.9 ± 0.458 mm[*A]	37°C, 24h	-	[4]
Botrytis cinerea	*Dictyopteris polypodioides*	Methanol	-	Flavonoid/Phenolic compounds/sesquiterpenes can be responsible for the antifungal activity	-	10 mm	24h	-	[5]

**Disc/Well Diffusion technique**									
				(present in brown algae)					
Colletotrichum acutatum	*Dictyota dichotoma* (June, fertile)	Hexane	-	-	500 µg/disc	15 mm	28°C	Graphical values	[6]
	Dictyota dichotoma (January, vegetative)	Hexane	-	-	500 µg/disc	16 mm	28°C	Graphical values	[6]
	Dictyota dichotoma (July, end of fertile)	Hexane	-	-	500 µg/disc	24 mm	28°C	Graphical values	[6]
	Dictyota dichotoma (June, fertile)	Hexane	-	-	500 µg/disc	17 mm	28°C	Graphical values	[6]
	Dictyota dichotoma (Septembre, vegetative)	Hexane	-	-	500 µg/disc	22 mm	28°C	Graphical values	[6]
	Dictyota implexa (July, fertile)	Hexane	-	-	500 µg/disc	24 mm	28°C	Graphical values	[6]
	Dictyota implexa (Novembre, vegetative)	Hexane	-	-	500 µg/disc	25 mm	28°C	Graphical values	[6]
	Dictyota implexa (Septembre, fertile)	Hexane	-	-	500 µg/disc	17 mm	28°C	Graphical values	[6]
	Dictyota spiralis (May, fertile)	Hexane	-	-	500 µg/disc	14 mm	28°C	Graphical values	[6]
Colletotrichum gloeosporioides	*Hypnea musciformis*	Dichloromethane: methanol (2:1)	Mannitol, octadecanoic acid and galactoglycerol	-	5 µg/mL	56.73%	28°C, 6 days	-	[7]
	Laurencia dendroidea	Dichloromethane: methanol (2:1)	Nonadecenoic acid	-	5 µg/mL	98.37%	28°C, 6 days	-	[7]
		Dichloromethane: methanol (2:1)	Hexadecanoic acid	-	5 µg/mL	100%	28°C, 6 days	-	[7]
	Pterocladiella capillacea	Dichloromethane: methanol (2:1)	Hexadecanoic acid, cholesterol and quercetin	-	5 µg/mL	58.91%	28°C, 6 days	-	[7]
Fusarium graminearum	*Dictyopteris polypodioides*	Methanol	-	Flavonoid/Phenolic compounds/sesquiterpenes can be responsible for the antifungal activity (present in brown algae)	-	8 mm	24h	-	[5]
Fusarium oxysporum	*Asparagopsis taxiformis*	Methanol	Chemical constituints of the main active fraction: 4,5-dimethyl-1H-pyrrole-2-carboxylic acid ethyl ester (56.012%), fatty acids, 14-methyl-pentadecanoic acid methyl ester (26.6%),	-	10 mg/mL	13 ± 1.7 mm	30°C, 72h	-	[2]

Disc/Well Diffusion technique

			octadecanoic acid methyl ester (8.46%), octadec-9-enoic acid 2,3-dihydroxy-propyl ester (4.11%), 9-octadecanoic acid, methyl ester (4.535%) and trace amount of chlorobenzene (0.09%).						
	Dictyopteris polypodioides	Methanol	-	Flavonoid/Phenolic compounds/sesquiterpenes can be responsible for the antifungal activity (present in brown algae).	-	9 mm	24h	-	[5]
	Padina boergesenii	Acetone	-	-	300 mg/mL	12.6 mm	$28^{o}C$, 72h	-	[8]
		Methanol	Hexadecanoic acid, methyl ester (50.32%), 9-octadecenoic acid, methyl ester (31.7%), tetradecanoic acid, methyl ester (18.4%), cis-11-Eicosenoic acid, methyl ester (15.7%)	-	300 mg/mL	11 mm	$28^{o}C$, 72h	Graphical result	[8]
	Polycladia indica	Ethanol	-	-	6 mg/disc	6 mm	5 days	-	[9]
	Polycladia myrica	Acetone	-	-	300 mg/mL	12 mm	$28^{o}C$, 72h	Graphical result	[8]
		Methanol	3,7-dimethylocta-1,6-dien-3-ol(80.32%), benzene, 1-methoxy-4-(2-propenyl) (77.7%), 3,7-dimethylocta-2,6-dienal (73.5%), 4,6,6-trimethyl bicyclo [3.1.1] hept-3-en-2-ol (67.3%), hexadecanoic acid, methyl ester (43.8%), octadecanoic acid, methyl ester (27.7%) and, 9-octadecenoic acid, methyl ester (21%)	-	300 mg/mL	15.3 mm	$28^{o}C$, 72h	-	[8]
	Sargassum cinereum	Acetone	-	-	300 mg/mL	13 mm	$28^{o}C$, 72h	Graphical result	[8]
		Methanol	3,7-dimethylocta-2,6-dienal (89.5%), 4,6,6-trimethyl bicyclo [3.1.1] hept-3-en-2-ol (65.42%), 3-Carene (59.6%) and hexadecanoic acid, methyl ester (13.8%)	-	300 mg/mL	14.6 mm	$28^{o}C$, 72h	-	[8]

**Disc/Well Diffusion technique**									
	Sargassum ilicifolium	Ethanol	-	Polyunsaturated esters (present in *Sargassum* species) may be the compound responsible for antimicrobial activity	4 mg/disc	8 mm	5 days	-	[9]
		Ethanol	-		6 mg/disc	10 mm	5 days	-	[9]
	Spatoglossum asperum	Oily fraction (2% chloroform + hexane)	Mixture of fatty acid esters	-	20 µg/disc	6 mm	28°C	Method modified (Variations of well-diffusion assay)	[10]
Fusarium oxysporum albedinis	*Dictyota dichotoma* (February, vegetative)	Hexane	-	-	500 µg/disc	8 mm	28°C	Graphical values	[6]
	Dictyota dichotoma (January, vegetative)	Hexane	-	-	500 µg/disc	9 mm	28°C	Graphical values	[6]
	Dictyota dichotoma (July, end of fertile)	Hexane	-	-	500 µg/disc	12 mm	28°C	Graphical values	[6]
	Dictyota dichotoma (June, fertile)	Hexane	-	-	500 µg/disc	12 mm	28°C	Graphical values	[6]
	Dictyota dichotoma (June, fertile)	Hexane	-	-	500 µg/disc	11 mm	28°C	Graphical values	[6]
	Dictyota dichotoma (Septembre, vegetative)	Hexane	-	-	500 µg/disc	10 mm	28°C	Graphical values	[6]
	Dictyota implexa (July, fertile)	Hexane	-	-	500 µg/disc	11 mm	28°C	Graphical values	[6]
	Dictyota implexa (June, fertile)	Hexane	-	-	500 µg/disc	10 mm	28°C	Graphical values	[6]
	Dictyota implexa (Novembre, vegetative)	Hexane	-	-	500 µg/disc	12 mm	28°C	Graphical values	[6]
	Dictyota implexa (Septembre, fertile)	Hexane	-	-	500 µg/disc	13 mm	28°C	Graphical values	[6]
	Dictyota spiralis (August, vegetative)	Hexane	-	-	500 µg/disc	8 mm	28°C	Graphical values	[6]
	Dictyota spiralis (June, fertile)	Hexane	-	-	500 µg/disc	10 mm	28°C	Graphical values	[6]
	Dictyota spiralis (May, fertile)	Hexane	-	-	500 µg/disc	11 mm	28°C	Graphical values	[6]
Fusarium oxysporum dianthi	*Dictyota dichotoma* (January, vegetative)	Hexane	-	-	500 µg/disc	10 mm	28°C	Graphical values	[6]
	Dictyota dichotoma (July, end of fertile)	Hexane	-	-	500 µg/disc	13 mm	28°C	Graphical values	[6]

**Disc/Well Diffusion technique**									
	Dictyota dichotoma (June, fertile)	Hexane	-	-	500 µg/disc	12 mm	28°C	Graphical values	[6]
	Dictyota dichotoma (June, fertile)	Hexane	-	-	500 µg/disc	13 mm	28°C	Graphical values	[6]
	Dictyota dichotoma (Septembre, vegetative)	Hexane	-	-	500 µg/disc	10 mm	28°C	Graphical values	[6]
	Dictyota implexa (July, fertile)	Hexane	-	-	500 µg/disc	14 mm	28°C	Graphical values	[6]
	Dictyota implexa (Novembre, vegetative)	Hexane	-	-	500 µg/disc	15 mm	28°C	Graphical values	[6]
	Dictyota implexa (Septembre, fertile)	Hexane	-	-	500 µg/disc	14 mm	28°C	Graphical values	[6]
	Dictyota spiralis (August, vegetative)	Hexane	-	-	500 µg/disc	9 mm	28°C	Graphical values	[6]
	Dictyota spiralis (June, fertile)	Hexane	-	-	500 µg/disc	10 mm	28°C	Graphical values	[6]
	Dictyota spiralis (May, fertile)	Hexane	-	-	500 µg/disc	10 mm	28°C	Graphical values	[6]
	Dictyota spiralis (Novembre, vegetative)	Hexane	-	-	500 µg/disc	12 mm	28°C	Graphical values	[6]
	Dictyota spiralis (October, vegetative)	Hexane	-	-	500 µg/disc	10 mm	28°C	Graphical values	[6]
Fusarium oxysporum lycopersici	*Dictyota dichotoma* (January, vegetative)	Hexane	-	-	500 µg/disc	12 mm	28°C	Graphical values	[6]
	Dictyota dichotoma (July, end of fertile)	Hexane	-	-	500 µg/disc	14 mm	28°C	Graphical values	[6]
	Dictyota dichotoma (June, fertile)	Hexane	-	-	500 µg/disc	9 mm	28°C	Graphical values	[6]
	Dictyota dichotoma (June, fertile)	Hexane	-	-	500 µg/disc	10 mm	28°C	Graphical values	[6]
	Dictyota dichotoma (Septembre, vegetative)	Hexane	-	-	500 µg/disc	15 mm	28°C	Graphical values	[6]
	Dictyota implexa (July, fertile)	Hexane	-	-	500 µg/disc	12 mm	28°C	Graphical values	[6]
	Dictyota implexa (Novembre, vegetative)	Hexane	-	-	500 µg/disc	15 mm	28°C	Graphical values	[6]
	Dictyota implexa (Septembre, fertile)	Hexane	-	-	500 µg/disc	9 mm	28°C	Graphical values	[6]

**Disc/Well Diffusion technique**									
	Dictyota spiralis (May, fertile)	Hexane	-	-	500 µg/disc	11 mm	28°C	Graphical values	[6]
Fusarium solani	*Spatoglossum asperum*	Oily fraction (2% chloroform + hexane)	Mixture of fatty acid esters	-	20 µg/disc	13 mm	28°C	Method modified (Variations of well-diffusion assay)	[10]
Fusarium sp.	*Anthophycus longifolius*	Benzene	Proteins and amino acids, flavonoids, phenolic compounds, alkaloids, sugar/glucosides	-	Standard concentration	8.5 ± 0.289 mm	37°C, 24h	The authors suggested than "zones greater than 10mm are considered positive results"	[4]
	Anthophycus longifolius	Acetic acid	Proteins and amino acids, glycosides, phenolic compounds, carbohydrates, alkaloids, sugar/glucosides.	-	Standard concentration	8.133 ± 0.133 mm	37°C, 24h		
	Anthophycus longifolius	Hexane	Proteins and amino acids, flavonoids, phenolic compounds, carbohydrates, alkaloids, coumarine, sugar/glucosides.	-	Standard concentration	7.23 ± 0.145 mm	37°C, 24h		
	Anthophycus longifolius	DMSO	Proteins and amino acids, flavonoids, glycosides, phenolic compounds, alkaloids, sugar/glucosides.	-	Standard concentration	7.733 ± 0.371 mm	37°C, 24h		
	Anthophycus longifolius	Diethyl ether	Proteins and amino acid, phenolic compounds, alkaloids, coumarine, sugar/glucosides.	-	Standard concentration	10.73 ± 0.371 mm	37°C, 24h	The authors sugestted than "zones greater than 10mm are considered positive results"	[4]
	Anthophycus longifolius	Chloroform	Proteins and amino acids, flavonoids, glycosides, phenolic compounds, alkaloids, coumarine, sugar/glucosides.	-	Standard concentration	11.2 ± 0.115 mm	37°C, 24h		[4]
Geotrichum sp.	*Dictyopteris polypodioides*	Methanol	-	Flavonoid/ Phenolic compounds/ sesquiterpenes can be responsible for the antifungal activity (present in brown algae)	-	10 mm	24h	-	[5]
Macrophomina phaseolina	*Polycladia indica*	Ethanol	-	-	6 mg/disc	9 mm	5 days	-	[9]
	Sargassum ilicifolium	Ethanol	-	Polyunsaturated esters (present in *Sargassum* species) may be the	6 mg/disc	10 mm	5 days	-	

Disc/Well Diffusion technique									
				compound responsible for antimicrobial activity					
	Spatoglossum asperum	Chloroform	-	-	6 mg/disc	3 mm	28°C	Method modified (Variations of well-diffusion assay)	[10]
		Methanol	-	-	6 mg/disc	4 mm	28°C		
		Oily fraction (2% chloroform + hexane)	Mixture of fatty acid esters	-	20 µg/disc	6 mm	28°C		
Penicillium sp.	*Dictyota dichotoma*	Diethyl ether	-	-	-	5-10 mm	30°C, 3 days	Weak or negligible activity	[11]
	Ulva lactuca	Chloroform	-	-	-	5 mm	30°C, 3 days	Weak or negligible activity	
Phialophora cinerescens	*Dictyota dichotoma* (June, fertile)	Hexane	-	-	500 µg/disc	10 mm	28°C	Graphical values	[6]
	Dictyota dichotoma (June, fertile)	Hexane	-	-	500 µg/disc	8 mm	28°C	Graphical values	
	Dictyota implexa (Septembre, fertile)	Hexane	-	-	500 µg/disc	11 mm	28°C	Graphical values	
	Dictyota spiralis (May, fertile)	Hexane	-	-	500 µg/disc	8 mm	28°C	Graphical values	
Phoma tracheiphila	*Dictyota dichotoma* (August, vegetative)	Hexane	-	-	500 µg/disc	11 mm	28°C	Graphical values	[6]
	Dictyota dichotoma (July, end of fertile)	Hexane	-	-	500 µg/disc	8 mm	28°C	Graphical values	[6]
	Dictyota dichotoma (June, fertile)	Hexane	-	-	500 µg/disc	13 mm	28°C	Graphical values	
	Dictyota implexa (July, fertile)	Hexane	-	-	500 µg/disc	8 mm	28°C	Graphical values	
	Dictyota implexa (June, fertile)	Hexane	-	-	500 µg/disc	15 mm	28°C	Graphical values	
	Dictyota implexa (May, fertile)	Hexane	-	-	500 µg/disc	14 mm	28°C	Graphical values	
	Dictyota implexa (Septembre, fertile)	Hexane	-	-	500 µg/disc	16 mm	28°C	Graphical values	
	Dictyota spiralis (August, vegetative)	Hexane	-	-	500 µg/disc	8 mm	28°C	Graphical values	
	Dictyota spiralis (July, fertile)	Hexane	-	-	500 µg/disc	11 mm	28°C	Graphical values	
	Dictyota spiralis (June, fertile)	Hexane	-	-	500 µg/disc	11 mm	28°C	Graphical values	
	Dictyota spiralis (June, fertile)	Hexane	-	-	500 µg/disc	10 mm	28°C	Graphical values	
	Dictyota spiralis (May, fertile)	Hexane	-	-	500 µg/disc	12 mm	28°C	Graphical values	

**Disc/Well Diffusion technique**									
	Dictyota spiralis (October, vegetative)	Hexane	-	-	500 µg/disc	20 mm	28°C	Graphical values	
	Dictyota spiralis (Septembre, fertile)	Hexane	-	-	500 µg/disc	8 mm	28°C	Graphical values	
Rhizoctonia solani	*Spatoglossum asperum*	Oily fraction (2% chloroform + hexane)	Mixture of fatty acid esters	-	20 µg/disc	18 mm	28°C	Method modified (Variations of well-diffusion assay)	[10]
	Dictyota dichotoma	Chloroform:metha nol 1:1 (v/v)	-	-	5%	35,8 mm	28±2°C, 48h	-	[12]
		Chloroform:metha nol 1:1 (v/v)	-	-	10%	37,3 mm	28±2°C, 48h	-	
		Chloroform:metha nol 1:1 (v/v)	-	-	15%	40,1 mm	28±2°C, 48h	-	
		Chloroform:metha nol 1:1 (v/v)	-	-	20%	42,12 mm	28±2°C, 48h	-	
		Chloroform:metha nol 1:1 (v/v)	-	-	5%	31,5 mm	28±2°C, 10 days	-	
		Chloroform:metha nol 1:1 (v/v)	-	-	10%	33,13 mm	28±2°C, 10 days	-	
		Chloroform:metha nol 1:1 (v/v)	-	-	15%	39,66 mm	28±2°C, 10 days	-	
		Chloroform:metha nol 1:1 (v/v)	-	-	20%	44,15 mm	28±2°C, 10 days	-	
	Padina gymnospora	Chloroform:metha nol 1:1 (v/v)	-	-	5%	32 mm	28±2°C, 48h	-	[12]
		Chloroform:metha nol 1:1 (v/v)	-	-	10%	35,12 mm	28±2°C, 48h	-	
	Padina gymnospora	Chloroform:metha nol 1:1 (v/v)	-	-	15%	37,33 mm	28±2°C, 48h	-	
		Chloroform:metha nol 1:1 (v/v)	-	-	20%	39,41 mm	28±2°C, 48h	-	
		Chloroform:metha nol 1:1 (v/v)	-	-	5%	27,5 mm	28±2°C, 10 days	-	
		Chloroform:metha nol 1:1 (v/v)	-	-	10%	30,3 mm	28±2°C, 10 days	-	
		Chloroform:metha nol 1:1 (v/v)	-	-	15%	37,16 mm	28±2°C, 10 days	-	
		Chloroform:metha nol 1:1 (v/v)	-	-	20%	41,9 mm	28±2°C, 10 days	-	
	Sargassum muticum	Chloroform:metha nol 1:1 (v/v)	-	-	5%	29,8 mm	28±2°C, 48h	-	
		Chloroform:metha nol 1:1 (v/v)	-	-	10%	33,44 mm	28±2°C, 48h	-	

**Disc/Well Diffusion technique**									
		Chloroform:methanol 1:1 (v/v)	-	-	15%	32,13 mm	28±2°C, 48h	-	
		Chloroform:methanol 1:1 (v/v)	-	-	20%	36,33 mm	28±2°C, 48h	-	
		Chloroform:methanol 1:1 (v/v)	-	-	5%	25 mm	28±2°C, 10 days	-	
		Chloroform:methanol 1:1 (v/v)	-	-	10%	27,16 mm	28±2°C, 10 days	-	
		Chloroform:methanol 1:1 (v/v)	-	-	15%	33,15 mm	28±2°C, 10 days	-	
		Chloroform:methanol 1:1 (v/v)	-	-	20%	39,2 mm	28±2°C, 10 days	-	
	Sargassum tenerrimum	Chloroform:methanol 1:1 (v/v)	-	n-hexadecanoic acid; Geranyl isovalerate; Oleic acid; 17-Octadecynoic acid; 9,12,15-Octadecatrienoic acid, 2,3-dihydroxypropyl ester, (Z,Z,Z)-; trans-13-Octadecenoic acid; 11-Octadecenoic acid, methyl ester; 6,9,12,15-Docosatetraenoic acid, methyl ester; Gibberellic acid; Fenretinide; 9,10-Secocholesta-5,7,10(19)-triene-3,24,25-triol, (3β,5Z,7E)-; Cholestan-3-ol, 2-methylene-, (3β,5α)-	5%	18,15 mm	28±2°C, 48h	-	
		Chloroform:methanol 1:1 (v/v)	-		10%	23,55 mm	28±2°C, 48h	-	
		Chloroform:methanol 1:1 (v/v)	-		15%	28,2 mm	28±2°C, 48h	-	
		Chloroform:methanol 1:1 (v/v)	-		20%	30,2 mm	28±2°C, 48h	-	
		Chloroform:methanol 1:1 (v/v)	-		5%	19,6 mm	28±2°C, 10 days	-	
		Chloroform:methanol 1:1 (v/v)	-		10%	21,9 mm	28±2°C, 10 days	-	
	Sargassum tenerrimum	Chloroform:methanol 1:1 (v/v)	-		15%	23,8 mm	28±2°C, 10 days	-	
		Chloroform:methanol 1:1 (v/v)	-		20%	29,5 mm	28±2°C, 10 days	-	[12]
	Sargassum wightii	Chloroform:methanol 1:1 (v/v)	-	-	5%	25,8 mm	28±2°C, 48h	-	
		Chloroform:methanol 1:1 (v/v)	-	-	10%	27,63 mm	28±2°C, 48h	-	
		Chloroform:methanol 1:1 (v/v)	-	-	15%	29,47 mm	28±2°C, 48h	-	

**Disc/Well Diffusion technique**									
		Chloroform:methanol 1:1 (v/v)	-	-	20%	32,72 mm	28±2°C, 48h	-	
		Chloroform:methanol 1:1 (v/v)	-	-	5%	21,9 mm	28±2°C, 10 days	-	
		Chloroform:methanol 1:1 (v/v)	-	-	10%	24,15 mm	28±2°C, 10 days	-	
		Chloroform:methanol 1:1 (v/v)	-	-	15%	28,32 mm	28±2°C, 10 days	-	
		Chloroform:methanol 1:1 (v/v)	-	-	20%	33,33 mm	28±2°C, 10 days	-	
Trichoderma sp.	*Asparagopsis taxiformis*	Methanol	Chemical constituents of the main active fraction: 4,5-dimethyl-1H-pyrrole-2-carboxylic acid ethyl ester (56.012%), fatty acids, 14-methyl-pentadecanoic acid methyl ester (26.6%), octadecanoic acid methyl ester (8.46%), octadec-9-enoic acid 2,3-dihydroxy-propyl ester (4.11%), 9-octadecanoic acid, methyl ester (4.535%) and trace amount of chlorobenzene (0.09%).	-	10 mg/mL	12 ± 1.9 mm	30°C, 72h	-	[2]
Verticillium dahliae	*Dictyopteris polypodioides*	Methanol	-	Flavonoid/ Phenolic compounds/ sesquiterpenes can be responsible for the antifungal activity (present in brown algae)	-	10 mm	24h	-	[5]

*[A]- the data analysis performed by the authors considered that “the greater values of 10 mm are positive results”.

Table S2. Data available about the antifungal activity against phytopathogenic fungi from macroalgae using the modified diffusion technique.

Modified Diffusion technique*B									
**Phytopathogenic Fungi**	**Macroalgae source**	**Extraction solvent**	**Extract composition (bibliographic information)**	**Extract concentration (mg/mL)**	**Algal dose (µL/petri)**	**Fungal growth (mm)**	**Control (mm)**	**Incubation conditions**	**Reference**
Aspergillus niger	*Ulva lactuca*	Methanol (Soxhlet extraction)	Rich in carbohydrates (43.91%), protein (28.85%), Ash (27.23%), Moisture (14.26%) and lipids (5.02%)	10 mg/mL	20 µL/Petri<br>40 µL/Petri<br>60 µL/Petri	59.25±1.06<br>54.00±11.31<br>47.00±0.70	61.00±0.00	25±2°C, 3 days	[13]
				50 mg/mL	40 µL/Petri<br>60 µL/Petri	53.00±15.50<br>46.50±3.53			
				100 mg/mL	20 µL/Petri<br>40 µL/Petri<br>60 µL/Petri	60.00±5.65<br>50.00±0.00<br>46.00±1.41			
				10 mg/mL	20 µL/Petri<br>40 µL/Petri<br>60 µL/Petri	73.00±2.82<br>57.50±14.84<br>47.00±0.70	80.00±0.00	25±2°C, 4 days	
				50 mg/mL	40 µL/Petri<br>60 µL/Petri	53.00±15.50<br>46.50±3.53			
				100 mg/mL	20 µL/Petri<br>40 µL/Petri<br>60 µL/Petri	62.00±8.48<br>50.00±0.00<br>46.00±1.41			
				10 mg/mL	20 µL/Petri<br>40 µL/Petri<br>60 µL/Petri	76.75±2.47<br>57.50±14.84<br>47.00±0.70	90.00±0.00	25±2°C, 5 days	
				50 mg/mL	20 µL/Petri<br>40 µL/Petri<br>60 µL/Petri	80.00±0.00<br>53.00±15.50<br>46.50±3.53			
				100 mg/mL	20 µL/Petri<br>40 µL/Petri<br>60 µL/Petri	62.00±8.48<br>50.00±0.00<br>46.00±1.41			
				10 mg/mL	20 µL/Petri<br>40 µL/Petri<br>60 µL/Petri	76.75±2.47<br>57.50±14.84<br>47.00±0.70	90.00±0.00	25±2°C, 6 days	
				50 mg/mL	20 µL/Petri<br>40 µL/Petri	80.00±0.00<br>53.00±15.50			

				**Modified Diffusion technique*[B]**					
					60 µL/Petri	46.50±3.53			
				100 mg/mL	20 µL/Petri	62.00±8.48			
					40 µL/Petri	50.00±0.00			
					60 µL/Petri	46.00±1.41			
Alternaria alternata	*Ulva lactuca*	Methanol (Soxhlet)	Rich in carbohydrates (43.91%), protein (28.85%), Ash (27.23%), Moisture (14.26%) and lipids (5.02%)	10 mg/mL	20 µL/Petri	21.00±2.12	24.00±0.00	25±2°C, 3 days	[13]
					40 µL/Petri	20.00±0.00			
					60 µL/Petri	21.00±1.41			
				50 mg/mL	20 µL/Petri	21.00±0.00			
					40 µL/Petri	20.00±0.00			
					60 µL/Petri	21.00±1.41			
				100 mg/mL	20 µL/Petri	22.50±0.70			
					40 µL/Petri	20.50±0.70			
					60 µL/Petri	20.50±0.70			
				10 mg/mL	20 µL/Petri	24.50±2.12	27.50±0.70	25±2°C, 4 days	
					40 µL/Petri	23.50±0.70			
					60 µL/Petri	24.00±1.41			
				50 mg/mL	20 µL/Petri	23.50±0.00			
					40 µL/Petri	23.00±0.70			
					60 µL/Petri	23.00±1.41			
				100 mg/mL	20 µL/Petri	26.50±3.53			
					40 µL/Petri	23.50±0.70			
					60 µL/Petri	26.00±1.41			
				10 mg/mL	20 µL/Petri	29.00±4.24	35.00±0.00	25±2°C, 5 days	
					40 µL/Petri	26.50±0.00			
					60 µL/Petri	25.50±0.70			
				50 mg/mL	20 µL/Petri	29.00±0.00			
					40 µL/Petri	26.25±1.76			
					60 µL/Petri	27.50±0.70			
				100 mg/mL	20 µL/Petri	30.00±7.07			
					40 µL/Petri	26.00±1.41			
					60 µL/Petri	27.00±1.41			
				1% (10 mg/mL)	20 µL/Petri	31.00±1.41	43.50±0.70	25±2°C, 6 days	
					40 µL/Petri	27.75±1.76			
					60 µL/Petri	28.00±0.41			
				50 mg/mL	20 µL/Petri	30.75±1.06			
					40 µL/Petri	27.00±1.41			
					60 µL/Petri	27.50±0.70			
				100 mg/mL	20 µL/Petri	30.50±6.36			
					40 µL/Petri	27.00±1.41			
					60 µL/Petri	27.00±1.41			
Penicillium expansum	*Ulva lactuca*	Methanol (Soxhlet)	Rich in carbohydrates (43.91%), protein (28.85%), Ash (27.23%), Moisture (14.26%) and lipids (5.02%)	10 mg/mL	20 µL/Petri	10.00 ±0.00	12.00±0.00	25±2°C, 3 days	[13]
					40 µL/Petri	9.75±1.06			
					60 µL/Petri	10.00 ±1.41			
				50 mg/mL	20 µL/Petri	10.50±0.35			

**Modified Diffusion technique*[B]**					
		40 µL/Petri	10.75 ±0.35		
		60 µL/Petri	9.75±1.06		
	100 mg/mL	20 µL/Petri	10.00±0.00		
		40 µL/Petri	11.00 ±0.00		
		60 µL/Petri	11.00 ±0.00		
	10 mg/mL	20 µL/Petri	13.00±0.70	16.00±0.70	25±2°C, 4 days
		40 µL/Petri	12.00±1.41		
		60 µL/Petri	12.75±1.76		
	50 mg/mL	20 µL/Petri	13.75±1.06		
		40 µL/Petri	13.00±0.70		
		60 µL/Petri	12.25±0.35		
	100 mg/mL	20 µL/Petri	12.50±0.70		
		40 µL/Petri	11.75±1.06		
		60 µL/Petri	12.00±1.41		
	10 mg/mL	20 µL/Petri	16.00±0.00	18.75±1.06	25±2°C, 5 days
		40 µL/Petri	14.25±0.35		
		60 µL/Petri	14.75±1.06		
	50 mg/mL	20 µL/Petri	15.50±0.70		
		40 µL/Petri	15.00±0.70		
		60 µL/Petri	14.25±0.35		
	100 mg/mL	20 µL/Petri	14.00±0.70		
		40 µL/Petri	14.00±0.00		
		60 µL/Petri	14.00±1.41		
	10 mg/mL	20 µL/Petri	18.75±0.35	22.25±1.76	25±2°C, 6 days
		40 µL/Petri	16.25±1.06		
		60 µL/Petri	15.50±0.70		
	50 mg/mL	20 µL/Petri	18.25±1.06		
		40 µL/Petri	15.25±0.35		
		60 µL/Petri	15.00±0.00		
	100 mg/mL	20 µL/Petri	17.75±0.35		
		40 µL/Petri	14.50±0.70		
		60 µL/Petri	14.50±0.70		

*[B] -Complete procedure description described by Vehapi et al. [13].

Table S3. Data available about the antifungal activity against phytopathogenic fungi from macroalgae using the poisoned food technique.

Poisoned food technique										
**Phytopathogenic Fungi**	**Macroalgae source**	**Extraction solvent**	**Extract composition**	**Extract composition proposed (bibliographic information)**	**Extract concentration (mg/mL)/ volume (mL)**	**Percentage of growth reduction (%)**	**Incubation conditions (Temperature)**	**Incubation conditions**	**Notes**	**Reference**
Colletotrichum falcatum	*Sargassum myricocystum**	Aqueous	-	-	5%	8.89	-	-	-	[14]
			-	-	10%	12.22	-	-	-	
		Ethanol	-	Lipophilic compounds	5%	33.33	-	-	-	
			-	Lipophilic compounds	10%	42.22	-	-	-	
	Hydropuntia edulis	Aqueous	-	-	5%	5.56	-	-	-	
		Aqueous	-	-	10%	11.11	-	-	-	
		Ethanol	-	-	5%	21.11	-	-	-	
		Ethanol	-	-	10%	36.67	-	-	-	
	Caulerpa racemosa	Aqueous	-	-	5%	3.33	-	-	-	
		Aqueous	-	-	10%	7.77	-	-	-	
		Ethanol	-	-	5%	16.66	-	-	-	
		Ethanol	-	-	10%	30	-	-	-	
Colletotrichum gloeosporioides	*Hypnea musciformis*	Dichloromethane:methanol (2:1)	Sesquiterpenes (Bibliographic comparison)	5 µg/mL	44.48 ± 0.09%	-	Field (algae source)	120 h	-	[15]
	Hypnea musciformis	Dichloromethane:methanol (2:1)	Mannitol, octadecanoic acid and galactoglycerol	-	45.48 ± 1.07%	-	Culture (algae source)	120 h	-	
	Kappaphycus alvarezii	Cold water	κ-carrageenan	-	0,50%	100	-	5 days	-	[16]
	Ochtodes secundiramea	Dichloromethane:methanol (2:1)	Monoterpenes (Bibliographic comparison)	-	84.52 ± 0.16%	-	Field (algae source)	120 h	-	[15]
	Ochtodes secundiramea	Dichloromethane:methanol (2:1)	Hexadecanoic acid	-	89.19 ± 1.64%	-	Culture (algae source)	120 h	-	
	Palisada flagellifera	Dichloromethane:methanol (2:1)	Sesquiterpenes (Bibliographic comparison)	-	25.58 ± 0.66%	-	Field (algae source)	120 h	-	
Fusarium oxysporum	*Calliblepharis floresii**	Aqueous	-	-	41.47 ± 1.56%	-	Culture (algae source)	5 days	Graphical value	[17]
		Methanol	-	-	2,2 mL	55	28 ± 2°C	5 days	Graphical value	
	Caulerpa chemnitzia	Aqueous	-	-	2,2 mL	50-55	28 ± 2°C	5 days	Graphical value	
		Methanol	-	-	2,2 mL	50-55	28 ± 2°C	5 days	Graphical value	
	Caulerpa racemosa	Aqueous	-	-	2,2 mL	65-70	28 ± 2°C	5 days	Graphical value	
		Methanol	-	-	2,2 mL	85-90	28 ± 2°C	5 days	Graphical value	[17]
		Aqueous	-	-	2,2 mL	50-55	28 ± 2°C	5 days	Graphical value	

**Poisoned food technique**										
	Caulerpa scalpelliformis	Methanol	-	-	2,2 mL	30-35	28 ± 2ºC	5 days	Graphical value	
	Caulerpa taxifolia	Aqueous	-	-	2,2 mL	55	28 ± 2ºC	5 days	Graphical value	
		Methanol	-	-	2,2 mL	50-55	28 ± 2ºC	5 days	Graphical value	
	Centroceras sp.	Aqueous	-	-	2,2 mL	40-45	28 ± 2ºC	5 days	Graphical value	
		Methanol	-	-	2,2 mL	45-50	28 ± 2ºC	5 days	Graphical value	
	Ceramium sp.	Aqueous	-	-	2,2 mL	55	28 ± 2ºC	5 days	Graphical value	
		Methanol	-	-	2,2 mL	55-60	28 ± 2ºC	5 days	Graphical value	
	Chaetomorpha antennina	Aqueous	-	-	2,2 mL	75-80	28 ± 2ºC	5 days	Graphical value	
		Methanol	-	-	2,2 mL	80-85	28 ± 2ºC	5 days	Graphical value	
	Codium indicum	Aqueous	-	-	2,2 mL	82	28 ± 2ºC	5 days	-	
		Methanol	-	-	2,2 mL	85-90	28 ± 2ºC	5 days	Graphical value	
	Polycladia indica	Aqueous	-	-	2,2 mL	55	28 ± 2ºC	5 days	Graphical value	
		Methanol	-	-	2,2 mL	60-65	28 ± 2ºC	5 days	Graphical value	
	Dictyota dicotoma	Aqueous	-	-	2,2 mL	50-55	28 ± 2ºC	5 days	Graphical value	
	Gelidium pulchrum	Aqueous	-	-	2,2 mL	50-55	28 ± 2ºC	5 days	Graphical value	
		Methanol	-	-	2,2 mL	60-65	28 ± 2ºC	5 days	Graphical value	
	Gracilaria corticata	Aqueous	-	-	2,2 mL	75-80	28 ± 2ºC	5 days	Graphical value	
		Methanol	-	-	2,2 mL	80-85	28 ± 2ºC	5 days	Graphical value	
	Halimeda tuna	Aqueous	-	-	2,2 mL	70-75	28 ± 2ºC	5 days	Graphical value	
		Methanol	-	-	2,2 mL	75-80	28 ± 2ºC	5 days	Graphical value	
	Halymenia porphyriformis	Aqueous	-	-	2,2 mL	35-40	28 ± 2ºC	5 days	Graphical value	
		Methanol	-	-	2,2 mL	40-45	28 ± 2ºC	5 days	Graphical value	
	Hypnea musciformis	Aqueous	-	-	2,2 mL	70-75	28 ± 2ºC	5 days	Graphical value	[17]
		Methanol	-	-	2,2 mL	55-60	28 ± 2ºC	5 days	Graphical value	
	Jania pedunculata var. *adhaerens*	Aqueous	-	-	2,2 mL	40-45	28 ± 2ºC	5 days	Graphical value	
		Methanol	-	-	2,2 mL	55	28 ± 2ºC	5 days	Graphical value	
	Jolyna laminariodes	Aqueous	-	-	2,2 mL	50	28 ± 2ºC	5 days	Graphical value	
		Methanol	-	-	2,2 mL	50-55	28 ± 2ºC	5 days	Graphical value	
	Osmundea pinnatifida	Aqueous	-	-	2,2 mL	35-40	28 ± 2ºC	5 days	Graphical value	
		Methanol	-	-	2,2 mL	40-45	28 ± 2ºC	5 days	Graphical value	
	Melanothamnus afaqhusainii	Aqueous	-	-	2,2 mL	60-65	28 ± 2ºC	5 days	Graphical value	
		Methanol	-	-	2,2 mL	65-70	28 ± 2ºC	5 days	Graphical value	
	Padina tetrastromatica	Aqueous	-	-	2,2 mL	86	28 ± 2ºC	5 days	-	
		Methanol	-	-	2,2 mL	95-100	28 ± 2ºC	5 days	Graphical value	
	Neoporphyra perforata	Aqueous	-	-	2,2 mL	60-65	28 ± 2ºC	5 days	Graphical value	
		Methanol	-	-	2,2 mL	65-70	28 ± 2ºC	5 days	Graphical value	
	Sargassum tenerrimum	Aqueous	-	-	2,2 mL	85-90	28 ± 2ºC	5 days	Graphical value	
		Methanol	-	-	2,2 mL	85-90	28 ± 2ºC	5 days	Graphical value	
	Sargassum aquifolium	Aqueous	-	-	2,2 mL	70-75	28 ± 2ºC	5 days	Graphical value	
		Methanol	-	-	2,2 mL	80-85	28 ± 2ºC	5 days	Graphical value	
	Sargassum wightii	Aqueous	-	-	2,2 mL	70-75	28 ± 2ºC	5 days	Graphical value	
		Methanol	-	-	2,2 mL	95-100	28 ± 2ºC	5 days	Graphical value	

**Poisoned food technique**										
	Scinaia huismanii	Aqueous	-	-	2,2 mL	55-60	28 ± 2°C	5 days	Graphical value	
		Methanol	-	-	2,2 mL	65-70	28 ± 2°C	5 days	Graphical value	
	*Steochospermum polypolides**	Aqueous	-	-	2,2 mL	50-55	28 ± 2°C	5 days	Graphical value	
		Methanol	-	-	2,2 mL	55	28 ± 2°C	5 days	Graphical value	
	Udotea sp.	Aqueous	-	-	2,2 mL	45-50	28 ± 2°C	5 days	Graphical value	[17]
		Methanol	-	-	2,2 mL	55	28 ± 2°C	5 days	Graphical value	
	Ulva rigida	Aqueous	-	-	2,2 mL	65-70	28 ± 2°C	5 days	Graphical value	
		Methanol	-	-	2,2 mL	70	28 ± 2°C	5 days	Graphical value	
	Valaniopsis sp.*	Aqueous	-	-	2,2 mL	45-50	28 ± 2°C	5 days	Graphical value	
		Methanol	-	-	2,2 mL	40-45	28 ± 2°C	5 days	Graphical value	
Ganoderma boninense	*Sargassum oligocystum*	Methanol	Cholest-5-en-3-ol, 24-propylidene-, (3á)-/ Hexadecanoic acid,2-hydroxy-1-(hydroxymethyl) ethyl ester/cis-Vaccenic acid/l-(+)-Ascorbic acid 2,6-dihexadecanoate/Hexadecanoic acid, methyl ester/Phenol, 2,4-bis(1,1-dimethylethyl)-	-	0,5 mg/mL	35	27 °C	7 days (daily measurements)	Graphical value	[18]
	Sargassum oligocystum	Chloroform	á-Tocopherol, O-methyl-/2H-1-Benzopyran-6-ol,3,4-dihydro-2,8-dimethyl-2-(4,8,12-trimethyl tridecyl)-, [2R-[2R*(4R*,8R*)]]-/Tetratriacontane/Di	-	0,5 mg/mL	30	27 °C	7 days (daily measurements)	Graphical value	
		Chloroform	isooctyl phthalate/l-(+)-Ascorbic acid 2,6-dihexadecanoate/Phytol/Tetradecanoic acid	-	1,0 mg/mL	40	27 °C	7 days (daily measurements)	Graphical value	
	Caulerpa racemosa	Methanol	Isobutyl methylphosphonofluoridate/9,12-Octadecadienoic acid	-	0,25 mg/mL	28,06	27 °C	7 days (daily measurements)	-	[18]

Poisoned food technique										
		Methanol	(Z,Z)-/Phytol/Benzenamine, 2-[2-(4-pyridinyl) ethyl]-/9,12,15-Octadecatrienoic acid, (Z,Z,Z)-	-	0,5 mg/mL	25	27 ^{o}C	7 days (daily measurements)	Graphical value	
		Methanol		-	1,0 mg/mL	15	27 ^{o}C	7 days (daily measurements)	Graphical value	
		Chloroform	á-Tocopherol, O-methyl-/Isobutyl methylphosphonofluoridate/Phytol/Benzenamine, 2-[2-(4-pyridinyl) ethyl]-/9,12,15-Octadecatrienoic acid, (Z,Z,Z)-/Tetradecanoic acid	-	0,25 mg/mL	20	27 ^{o}C	7 days (daily measurements)	Graphical value	
		Chloroform		-	0,5 mg/mL	40	27 ^{o}C	7 days (daily measurements)	Graphical value	
		Chloroform		-	1,0 mg/mL	35	27 ^{o}C	7 days (daily measurements)	Graphical value	
	Caulerpa lamourouxii	Methanol	Isobutyl methylphosphonofluoridate/1-Monolinoleoylglycerol trimethylsilyl ether/Spirost-8-en-11-one,3-hydroxy, (3á,5á,14á,20á,22á,25R)-/Benzenamine,2-[2-(4-pyridinyl)ethyl]-/Hexacyclo [7.2.2.2(4,7).0(3,8).0(12,14).0(13,15)pentadecan-1-ol-2-one/Ethyl iso-allocholate/l-(+)-Ascorbic acid 2,6-dihexadecanoate	-	0,25 mg/mL	24.50	27 ^{o}C	7 days (daily measurements)	-	
		Methanol		-	0,5 mg/mL	30	27 ^{o}C	7 days (daily measurements)	Graphical value	
		Methanol		-	1,0 mg/mL	30	27 ^{o}C	7 days (daily measurements)	Graphical value	

**Poisoned food technique**										
	Caulerpa lamourouxii	Chloroform	Furo[3',4':6,7]naphtho[2,3-d]-1,3-dioxol-6(5aH)-one,5,8,8a,9-tetrahydro-5-(3,4,5-trimethoxyphenyl)-,[5R-(5à,5aá,8aà)]-/(+)-ç-Tocopherol, O-methyl-/Isobutyl methylphosphonofluoridate/Cholesta-5,7,9(11)-trien-3-ol acetate/Hexadecanamide/Phytol/l-(+)-Ascorbic acid 2,6-dihexadecanoate	-	0,25 mg/mL	27.44	27 ^{o}C	7 days (daily measurements)	-	[18]
		Chloroform		-	0,5 mg/mL	30	27 ^{o}C	7 days (daily measurements)	Graphical value	
		Chloroform		-	1,0 mg/mL	35	27 ^{o}C	7 days (daily measurements)	Graphical value	
	Halimeda macrophysa	Methanol	Benzenamine,2-[2-(4-pyridinyl) ethyl]-/1-Monolinoleoylglycerol trimethylsilyl ether/Isobutyl methylphosphonofluoridate/l-(+)-ascorbic acid 2,6-dihexadecanoate	-	0,25 mg/mL	15	27 ^{o}C	7 days (daily measurements)	Graphical value	
	Halimeda macrophysa	Methanol		-	1,0 mg/mL	30	27 ^{o}C	7 days (daily measurements)	Graphical value	
		Chloroform	Cholesterol/2-[4-methyl-6-(2,6,6-trimethylcyclohex-1-enyl) hexa-1,3,5-trienyl] cyclohex-1-en-1-carboxaldehyde/Phytol/l-(+)-Ascorbic acid 2,6-dihexadecanoate/Isobutyl methylphosphonofluoridate/ç-Sitosterol/Benzenamine,2-[2-(4-pyridinyl)ethyl]-	-	0,25 mg/mL	25.74	27 ^{o}C	7 days (daily measurements)	-	
		Chloroform		-	0,5 mg/mL	20	27 ^{o}C	7 days (daily measurements)	Graphical value	
		Chloroform		-	1,0 mg/mL	30	27 ^{o}C	7 days (daily measurements)	Graphical value	

**Poisoned food technique**										
	Sargassum oligocystum	Methanol	Cholest-5-en-3-ol, 24-propylidene-, (3á)-/ Hexadecanoic acid,2-hydroxy-1-(hydroxymethyl) ethyl ester/cis-Vaccenic acid/l-(+)-Ascorbic acid 2,6-dihexadecanoate/Hexadecanoic acid, methyl ester/Phenol, 2,4-bis(1,1-dimethylethyl)-	-	0,5 mg/mL	28	27 ^{o}C	7 days (daily measurements)	Graphical value	[18]
Macrophomina phaseolina	*Calliblepharis floresii**	Methanol	-	-	2,2 mL	30-35	28 ± 2°C	5 days	Graphical value	[17]
	Caulerpa racemosa	Aqueous	-	-	2,2 mL	55-60	28 ± 2°C	5 days	Graphical value	
		Methanol	-	-	2,2 mL	60-65	28 ± 2°C	5 days	Graphical value	
	Caulerpa taxifolia	Aqueous	-	-	2,2 mL	45-50	28 ± 2°C	5 days	Graphical value	
		Methanol	-	-	2,2 mL	50-55	28 ± 2°C	5 days	Graphical value	
	Centroceras sp.	Aqueous	-	-	2,2 mL	35-40	28 ± 2°C	5 days	Graphical value	
		Methanol	-	-	2,2 mL	40-45	28 ± 2°C	5 days	Graphical value	
	Ceramium sp.	Methanol	-	-	2,2 mL	65-70	28 ± 2°C	5 days	Graphical value	
	Chaetomorpha antennina	Aqueous	-	-	2,2 mL	25-30	28 ± 2°C	5 days	Graphical value	
		Methanol	-	-	2,2 mL	30-35	28 ± 2°C	5 days	Graphical value	
	Codium indicum	Aqueous	-	-	2,2 mL	40-45	28 ± 2°C	5 days	Graphical value	
		Methanol	-	-	2,2 mL	50-55	28 ± 2°C	5 days	Graphical value	
	Polycladia indica	Aqueous	-	-	2,2 mL	40-45	28 ± 2°C	5 days	Graphical value	
		Methanol	-	-	2,2 mL	45-50	28 ± 2°C	5 days	Graphical value	
	Dictyota dicotoma	Aqueous	-	-	2,2 mL	45-50	28 ± 2°C	5 days	Graphical value	
		Methanol	-	-	2,2 mL	50	28 ± 2°C	5 days	Graphical value	
	Gelidium pulchrum	Methanol	-	-	2,2 mL	75-80	28 ± 2°C	5 days	Graphical value	
	Gracilaria corticata	Aqueous	-	-	2,2 mL	65-70	28 ± 2°C	5 days	Graphical value	
		Methanol	-	-	2,2 mL	75-80	28 ± 2°C	5 days	Graphical value	
	Halymenia porphyriformis	Methanol	-	-	2,2 mL	45-50	28 ± 2°C	5 days	Graphical value	
	Hypnea musciformis	Aqueous	-	-	2,2 mL	55-60	28 ± 2°C	5 days	Graphical value	
		Methanol	-	-	2,2 mL	60-65	28 ± 2°C	5 days	Graphical value	
	Jania pedunculata var. *adhaerens*	Methanol	-	-	2,2 mL	40-45	28 ± 2°C	5 days	Graphical value	
	Jolyna laminariodes	Aqueous	-	-	2,2 mL	40-45	28 ± 2°C	5 days	Graphical value	
	Jolyna laminariodes	Methanol	-	-	2,2 mL	45	28 ± 2°C	5 days	Graphical value	[17]

**Poisoned food technique**										
	Osmundea pinnatifida	Methanol	-	-	2,2 mL	20-25	28 ± 2ºC	5 days	Graphical value	
	Melanothamnus afaqhusainii	Aqueous	-	-	2,2 mL	30-35	28 ± 2ºC	5 days	Graphical value	
		Methanol	-	-	2,2 mL	30-35	28 ± 2ºC	5 days	Graphical value	
	Padina tetrastromatica	Aqueous	-	-	2,2 mL	45-50	28 ± 2ºC	5 days	Graphical value	
		Methanol	-	-	2,2 mL	45-50	28 ± 2ºC	5 days	Graphical value	
	Neoporphyra perforata	Methanol	-	-	2,2 mL	30-35	28 ± 2ºC	5 days	Graphical value	
	Sargassum tenerrimum	Aqueous	-	-	2,2 mL	86	28 ± 2ºC	5 days	-	
		Methanol	-	-	2,2 mL	60-65	28 ± 2ºC	5 days	Graphical value	
	Sargassum aquifolium	Aqueous	-	-	2,2 mL	55-60	28 ± 2ºC	5 days	Graphical value	
		Methanol	-	-	2,2 mL	65-70	28 ± 2ºC	5 days	Graphical value	
	Sargassum wightii	Aqueous	-	-	2,2 mL	60	28 ± 2ºC	5 days	Graphical value	
		Methanol	-	-	2,2 mL	85-90	28 ± 2ºC	5 days	Graphical value	
	Scinaia huismanii	Methanol	-	-	2,2 mL	55	28 ± 2ºC	5 days	Graphical value	
	*Steochospermum polypolides**	Aqueous	-	-	2,2 mL	55-60	28 ± 2ºC	5 days	Graphical value	
		Methanol	-	-	2,2 mL	60-65	28 ± 2ºC	5 days	Graphical value	
	Udotea sp.	Aqueous	-	-	2,2 mL	45-50	28 ± 2ºC	5 days	Graphical value	
		Methanol	-	-	2,2 mL	50-55	28 ± 2ºC	5 days	Graphical value	
	Ulva rigida	Aqueous	-	-	2,2 mL	40-45	28 ± 2ºC	5 days	Graphical value	
		Methanol	-	-	2,2 mL	45	28 ± 2ºC	5 days	Graphical value	
	Valaniopsis sp.*	Aqueous	-	-	2,2 mL	60-65	28 ± 2ºC	5 days	Graphical value	
		Methanol	-	-	2,2 mL	65-70	28 ± 2ºC	5 days	Graphical value	
Rhizoctonia solani	*Calliblepharis floresii**	Methanol	-	-	2,2 mL	40-45	28 ± 2ºC	5 days	Graphical value	[17]
	Centroceras sp.	Methanol	-	-	2,2 mL	30-35	28 ± 2ºC	5 days	Graphical value	
	Ceramium sp.	Methanol	-	-	2,2 mL	50-55	28 ± 2ºC	5 days	Graphical value	
	Chaetomorpha antennina	Aqueous	-	-	2,2 mL	40-45	28 ± 2ºC	5 days	Graphical value	
		Methanol	-	-	2,2 mL	50	28 ± 2ºC	5 days	Graphical value	
	Codium indicum	Aqueous	-	-	2,2 mL	40	28 ± 2ºC	5 days	Graphical value	
		Methanol	-	-	2,2 mL	45-50	28 ± 2ºC	5 days	Graphical value	
	Polycladia indica	Methanol	-	-	2,2 mL	30-35	28 ± 2ºC	5 days	Graphical value	
	Gelidium pulchrum	Methanol	-	-	2,2 mL	45-50	28 ± 2ºC	5 days	Graphical value	
	Gracilaria corticata	Aqueous	-	-	2,2 mL	30-35	28 ± 2ºC	5 days	Graphical value	
		Methanol	-	-	2,2 mL	30-35	28 ± 2ºC	5 days	Graphical value	
	Halymenia porphyriformis	Methanol	-	-	2,2 mL	45-50	28 ± 2ºC	5 days	Graphical value	
	Hypnea musciformis	Aqueous	-	-	2,2 mL	40-45	28 ± 2ºC	5 days	Graphical value	
		Methanol	-	-	2,2 mL	40	28 ± 2ºC	5 days	Graphical value	
	Jania pedunculata var. *adhaerens*	Methanol	-	-	2,2 mL	40-45	28 ± 2ºC	5 days	Graphical value	
	Osmundea pinnatifida	Methanol	-	-	2,2 mL	25-30	28 ± 2ºC	5 days	Graphical value	[17]

**Poisoned food technique**										
	Melanothamnus afaqhusainii	Methanol	-	-	2,2 mL	65-70	28 ± 2°C	5 days	Graphical value	
	Padina tetrastromatica	Aqueous	-	-	2,2 mL	45-50	28 ± 2°C	5 days	Graphical value	
		Methanol	-	-	2,2 mL	50-55	28 ± 2°C	5 days	Graphical value	
	Neoporphyra perforata	Methanol	-	-	2,2 mL	40-45	28 ± 2°C	5 days	Graphical value	
	Sargassum tenerrimum	Aqueous	-	-	2,2 mL	74	28 ± 2°C	5 days	-	
		Methanol	-	-	2,2 mL	45-50	28 ± 2°C	5 days	Graphical value	
	Sargassum aquifolium	Aqueous	-	-	2,2 mL	45	28 ± 2°C	5 days	Graphical value	
		Methanol	-	-	2,2 mL	45	28 ± 2°C	5 days	Graphical value	
	Sargassum wightii	Aqueous	-	-	2,2 mL	40-45	28 ± 2°C	5 days	Graphical value	
		Methanol	-	-	2,2 mL	75-80	28 ± 2°C	5 days	Graphical value	
	*Steochospermum polypolides**	Aqueous	-	-	2,2 mL	55-60	28 ± 2°C	5 days	Graphical value	
		Methanol	-	-	2,2 mL	60-65	28 ± 2°C	5 days	Graphical value	
	Udotea sp.	Aqueous	-	-	2,2 mL	45-50	28 ± 2°C	5 days	Graphical value	
		Methanol	-	-	2,2 mL	50-55	28 ± 2°C	5 days	Graphical value	
	Ulva rigida	Aqueous	-	-	2,2 mL	25-30	28 ± 2°C	5 days	Graphical value	
		Methanol	-	-	2,2 mL	10-15	28 ± 2°C	5 days	Graphical value	
	Valaniopsis sp.*	Aqueous	-	-	2,2 mL	60	28 ± 2°C	5 days	Graphical value	
		Methanol	-	-	2,2 mL	60-65	28 ± 2°C	5 days	Graphical value	

*- Species not found in AlgaeBase database (https://www.algaebase.org/).

Table S4: Data available about the antifungal activity against phytopathogenic fungi from macroalgae using the poisoned food technique (data expressed in mycelial growth).

Poisoned food technique (data expressed in mycelial growth)								
**Phytopathogenic Fungi**	**Macroalgae source**	**Solvent in the extraction**	**Extract composition proposed (bibliographic information)**	**Concentration of algae extract (%)**	**Mycelial growth (mm)**	**Control (mm)**	**Incubation period**	**References**
Fusarium oxysporum f.sp. *udum*	*Sargassum myricocystum**	Alcohol	The presence of flavonoid and phenolic compounds could be the reason of antifungal activity in brown seaweeds	10%	39,4	48,5	24h	[19]
		Alcohol		15%	38,2	48,5	24h	
		Alcohol		20%	35,3	48,5	24h	
		Alcohol		25%	30,1	48,5	24h	
		Alcohol		30%	28,1	48,5	24h	
		Alcohol		10%	49,7	62,4	48h	
		Alcohol		15%	45,7	62,4	48h	
		Alcohol		20%	36,8	62,4	48h	
		Alcohol		25%	34,7	62,4	48h	
		Alcohol		30%	33,9	62,4	48h	
		Alcohol		10%	54,2	81,9	72h	
		Alcohol		15%	49,4	81,9	72h	
		Alcohol		20%	43,3	81,9	72h	
		Alcohol		25%	38,3	81,9	72h	
		Alcohol		30%	34,7	81,9	72h	
		Alcohol		10%	62,1	85,2	96h	
		Alcohol		15%	57,5	85,2	96h	
		Alcohol		20%	46,8	85,2	96h	
		Alcohol		25%	42,4	85,2	96h	
		Alcohol		30%	39,4	85,2	96h	
		Alcohol		10%	65,7	90,0	108h	
		Alcohol		15%	61,6	90,0	108h	
		Alcohol		20%	52,8	90,0	108h	
		Alcohol		25%	45,8	90,0	108h	
		Alcohol		30%	44,3	90,0	108h	
	Hydropuntia edulis	Alcohol	-	10%	43,7	48,5	24h	[19]
		Alcohol	-	15%	40,5	48,5	24h	
		Alcohol	-	20%	37,3	48,5	24h	
		Alcohol	-	25%	36,4	48,5	24h	
		Alcohol	-	30%	33,2	48,5	24h	
		Alcohol	-	10%	53,4	62,4	48h	
		Alcohol	-	15%	47,1	62,4	48h	
		Alcohol	-	20%	42,8	62,4	48h	
		Alcohol	-	25%	39,8	62,4	48h	
	Hydropuntia edulis	Alcohol	-	30%	38,1	62,4	48h	
		Alcohol	-	10%	58,8	81,9	72h	
		Alcohol	-	15%	56,5	81,9	72h	
		Alcohol	-	20%	47,7	81,9	72h	

Poisoned food technique (data expressed in mycelial growth)								
		Alcohol	-	25%	44,9	81,9	72h	
		Alcohol	-	30%	40,5	81,9	72h	[19]
		Alcohol	-	10%	69,3	85,2	96h	
		Alcohol	-	15%	60,7	85,2	96h	
		Alcohol	-	20%	55,1	85,2	96h	
		Alcohol	-	25%	53,6	85,2	96h	
		Alcohol	-	30%	47,6	85,2	96h	
		Alcohol	-	10%	73,8	90,0	108h	
		Alcohol	-	15%	67,2	90,0	108h	
		Alcohol	-	20%	62	90,0	108h	
		Alcohol	-	25%	59,4	90,0	108h	
		Alcohol	-	30%	53,1	90,0	108h	
	Caulerpa racemosa	Alcohol	-	10%	43	48,5	24h	[19]
		Alcohol	-	15%	41,5	48,5	24h	
		Alcohol	-	20%	40,2	48,5	24h	
		Alcohol	-	25%	39	48,5	24h	
		Alcohol	-	30%	37,7	48,5	24h	
		Alcohol	-	10%	58,1	62,4	48h	
		Alcohol	-	15%	50,4	62,4	48h	
		Alcohol	-	20%	44,7	62,4	48h	
		Alcohol	-	25%	41	62,4	48h	
		Alcohol	-	30%	39,7	62,4	48h	
		Alcohol	-	10%	62,7	81,9	72h	
		Alcohol	-	15%	55,7	81,9	72h	
		Alcohol	-	20%	51,3	81,9	72h	
		Alcohol	-	25%	45,8	81,9	72h	
		Alcohol	-	30%	44,7	81,9	72h	
		Alcohol	-	10%	72,6	85,2	96h	
		Alcohol	-	15%	63,8	85,2	96h	
		Alcohol	-	20%	58,7	85,2	96h	
		Alcohol	-	25%	54	85,2	96h	
		Alcohol	-	30%	50,7	85,2	96h	
		Alcohol	-	10%	79,2	90,0	108h	
		Alcohol	-	15%	71,2	90,0	108h	
		Alcohol	-	20%	68,3	90,0	108h	
	Caulerpa racemosa	Alcohol	-	25%	61,6	90,0	108h	
		Alcohol	-	30%	58,3	90,0	108h	

*- Species not found in AlgaeBase database (https://www.algaebase.org/).

Table S5. Data available about the antifungal activity against phytopathogenic fungi from macroalgae by the evaluation of macroconidia germination.

Macroconidia germination								
**Phytopathogenic Fungi**	**Macroalgae source**	**Extraction solvent**	**Extract concentration**	**Results (% of germinated conidia)**	**Macroconidia concentration (Inoculum initial)**	**Incubation conditions**	**Notes**	**Reference**
Fusarium culmorum DEM Fc37	*Fucus vesiculosus*	Supercritical fluid extraction (CO2)	0.05% (12.5 µL)	60-70%	2.0 × 10^6/mL	20°C, 24h (darkness)	Graphical data	[20]
			0.05% (12.5 µL)	60-80%	2.0 × 10^6/mL	20°C, 48h (darkness)	Graphical data	
			0.05% (12.5 µL)	60-70%	2.0 × 10^6/mL	20°C, 72h (darkness)	Graphical data	
			0.05% (12.5 µL)	60-80%	2.0 × 10^6/mL	20°C, 96h (darkness)	Graphical data	
			0.05% (12.5 µL)	70-80%	2.0 × 10^6/mL	20°C, 120h (darkness)	Graphical data	
			0.05% (12.5 µL)	80-90%	2.0 × 10^6/mL	20°C, 144h (darkness)	Graphical data	
			0.2% (50 µL)	40-50%	2.0 × 10^6/mL	20°C, 24h (darkness)	Graphical data	
			0.2% (50 µL)	40-50%	2.0 × 10^6/mL	20°C, 48h (darkness)	Graphical data	
			0.2% (50 µL)	40-60%	2.0 × 10^6/mL	20°C, 72h (darkness)	Graphical data	
			0.2% (50 µL)	40-50%	2.0 × 10^6/mL	20°C, 96h (darkness)	Graphical data	
			0.2% (50 µL)	50-60%	2.0 × 10^6/mL	20°C, 120h (darkness)	Graphical data	
			0.2% (50 µL)	60%	2.0 × 10^6/mL	20°C, 144h (darkness)	Graphical data	
			0.5% (125 µL)	0-10%	2.0 × 10^6/mL	20°C, 24h (darkness)	Graphical data	
			0.5% (125 µL)	0-10%	2.0 × 10^6/mL	20°C, 48h (darkness)	Graphical data	
			0.5% (125 µL)	0%	2.0 × 10^6/mL	20°C, 72h (darkness)	Graphical data	
			0.5% (125 µL)	0%	2.0 × 10^6/mL	20°C, 96h (darkness)	Graphical data	
			0.5% (125 µL)	0%	2.0 × 10^6/mL	20°C, 120h (darkness)	Graphical data	
			0.5% (125 µL)	0%	2.0 × 10^6/mL	20°C, 144h (darkness)	Graphical data	
			0.05% (12.5 µL)	60-70%	2.0 × 10^6/mL	20°C, 24h (darkness)	Graphical data	
			0.05% (12.5 µL)	70-80%	2.0 × 10^6/mL	20°C, 48h (darkness)	Graphical data	
	Fucus vesiculosus	Supercritical fluid extraction (CO2)	0.05% (12.5 µL)	60-70%	2.0 × 10^6/mL	20°C, 72h (darkness)	Graphical data	
			0.05% (12.5 µL)	60%	2.0 × 10^6/mL	20°C, 96h (darkness)	Graphical data	
			0.05% (12.5 µL)	60-70%	2.0 × 10^6/mL	20°C, 120h (darkness)	Graphical data	
			0.05% (12.5 µL)	70-80%	2.0 × 10^6/mL	20°C, 144h (darkness)	Graphical data	
			0.2% (50 µL)	30-40%	2.0 × 10^6/mL	20°C, 24h (darkness)	Graphical data	
			0.2% (50 µL)	30-40%	2.0 × 10^6/mL	20°C, 48h (darkness)	Graphical data	
			0.2% (50 µL)	40%	2.0 × 10^6/mL	20°C, 72h (darkness)	Graphical data	
			0.2% (50 µL)	40-50%	2.0 × 10^6/mL	20°C, 96h (darkness)	Graphical data	
			0.2% (50 µL)	40-50%	2.0 × 10^6/mL	20°C, 120h (darkness)	Graphical data	
			0.2% (50 µL)	40-50%	2.0 × 10^6/mL	20°C, 144h (darkness)	Graphical data	
			0.5% (125 µL)	0%	2.0 × 10^6/mL	20°C, 24h (darkness)	Graphical data	
			0.5% (125 µL)	0%	2.0 × 10^6/mL	20°C, 48h (darkness)	Graphical data	
			0.5% (125 µL)	0%	2.0 × 10^6/mL	20°C, 72h (darkness)	Graphical data	
			0.5% (125 µL)	0%	2.0 × 10^6/mL	20°C, 96h (darkness)	Graphical data	

**Macroconidia germination**								
			0.5% (125 µL)	0%	2.0 × 10^6/mL	20ºC, 120h (darkness)	Graphical data	
	Fucus vesiculosus	Supercritical fluid extraction (CO2)	0.5% (125 µL)	0%	2.0 × 10^6/mL	20ºC, 144h (darkness)	Graphical data	[20]
Fusarium culmorum FcCBS122	*Fucus vesiculosus*	Supercritical fluid extraction (CO2)	0.05% (12.5 µL)	40-50%	2.0 × 10^6/mL	20ºC, 24h (darkness)	Graphical data	[20]
			0.05% (12.5 µL)	40-60%	2.0 × 10^6/mL	20ºC, 48h (darkness)	Graphical data	
			0.05% (12.5 µL)	50-60%	2.0 × 10^6/mL	20ºC, 72h (darkness)	Graphical data	
			0.05% (12.5 µL)	60-80%	2.0 × 10^6/mL	20ºC, 96h (darkness)	Graphical data	
			0.05% (12.5 µL)	70-80%	2.0 × 10^6/mL	20ºC, 120h (darkness)	Graphical data	
			0.05% (12.5 µL)	80-90%	2.0 × 10^6/mL	20ºC, 144h (darkness)	Graphical data	
			0.2% (50 µL)	20-30%	2.0 × 10^6/mL	20ºC, 24h (darkness)	Graphical data	
			0.2% (50 µL)	20-40%	2.0 × 10^6/mL	20ºC, 48h (darkness)	Graphical data	
			0.2% (50 µL)	20-40%	2.0 × 10^6/mL	20ºC, 72h (darkness)	Graphical data	
			0.2% (50 µL)	30-40%	2.0 × 10^6/mL	20ºC, 96h (darkness)	Graphical data	
			0.2% (50 µL)	30-40%	2.0 × 10^6/mL	20ºC, 120h (darkness)	Graphical data	
			0.2% (50 µL)	40-50%	2.0 × 10^6/mL	20ºC, 144h (darkness)	Graphical data	
			0.5% (125 µL)	0-10%	2.0 × 10^6/mL	20ºC, 48h (darkness)	Graphical data	
			0.5% (125 µL)	20-30%	2.0 × 10^6/mL	20ºC, 72h (darkness)	Graphical data	
			0.5% (125 µL)	20-30%	2.0 × 10^6/mL	20ºC, 96h (darkness)	Graphical data	
			0.5% (125 µL)	20-40%	2.0 × 10^6/mL	20ºC, 120h (darkness)	Graphical data	
			0.5% (125 µL)	30-40%	2.0 × 10^6/mL	20ºC, 144h (darkness)	Graphical data	
			0.05% (12.5 µL)	80-90%	2.0 × 10^6/mL	20ºC, 24h (darkness)	Graphical data	
			0.05% (12.5 µL)	70-80%	2.0 × 10^6/mL	20ºC, 48h (darkness)	Graphical data	
			0.05% (12.5 µL)	60-70%	2.0 × 10^6/mL	20ºC, 72h (darkness)	Graphical data	
			0.05% (12.5 µL)	60-70%	2.0 × 10^6/mL	20ºC, 96h (darkness)	Graphical data	
			0.05% (12.5 µL)	60-70%	2.0 × 10^6/mL	20ºC, 120h (darkness)	Graphical data	
			0.05% (12.5 µL)	70-80%	2.0 × 10^6/mL	20ºC, 144h (darkness)	Graphical data	
			0.2% (50 µL)	40-50%	2.0 × 10^6/mL	20ºC, 24h (darkness)	Graphical data	
			0.2% (50 µL)	40%	2.0 × 10^6/mL	20ºC, 48h (darkness)	Graphical data	
			0.2% (50 µL)	40%	2.0 × 10^6/mL	20ºC, 72h (darkness)	Graphical data	
			0.2% (50 µL)	30-40%	2.0 × 10^6/mL	20ºC, 96h (darkness)	Graphical data	
			0.2% (50 µL)	30-40%	2.0 × 10^6/mL	20ºC, 120h (darkness)	Graphical data	
			0.2% (50 µL)	40%	2.0 × 10^6/mL	20ºC, 144h (darkness)	Graphical data	
Fusarium oxysporum DEM Fo38	*Fucus vesiculosus*	Supercritical fluid extraction (CO2)	0.2% (50 µL)	30-40%	2.0 × 10^6/mL	20ºC, 48h (darkness)	Graphical data	[20]
			0.2% (50 µL)	40-50%	2.0 × 10^6/mL	20ºC, 72h (darkness)	Graphical data	
			0.2% (50 µL)	50%	2.0 × 10^6/mL	20ºC, 96h (darkness)	Graphical data	
			0.2% (50 µL)	60-70%	2.0 × 10^6/mL	20ºC, 120h (darkness)	Graphical data	
			0.2% (50 µL)	70-80%	2.0 × 10^6/mL	20ºC, 144h (darkness)	Graphical data	
			0.5% (125 µL)	20-30%	2.0 × 10^6/mL	20ºC, 72h (darkness)	Graphical data	
			0.5% (125 µL)	20-30%	2.0 × 10^6/mL	20ºC, 96h (darkness)	Graphical data	

			**Macroconidia germination**					
			0.5% (125 µL)	30-40%	2.0 × 10^6/mL	20°C, 120h (darkness)	Graphical data	
	Fucus vesiculosus	Supercritical fluid extraction (CO2)	0.5% (125 µL)	30-40%	2.0 × 10^6/mL	20°C, 144h (darkness)	Graphical data	[20]
			0.05% (12.5 µL)	40-50%	2.0 × 10^6/mL	20°C, 24h (darkness)	Graphical data	
			0.05% (12.5 µL)	60-70%	2.0 × 10^6/mL	20°C, 48h (darkness)	Graphical data	
			0.05% (12.5 µL)	50-60%	2.0 × 10^6/mL	20°C, 72h (darkness)	Graphical data	
			0.05% (12.5 µL)	60%	2.0 × 10^6/mL	20°C, 96h (darkness)	Graphical data	
			0.05% (12.5 µL)	60-70%	2.0 × 10^6/mL	20°C, 120h (darkness)	Graphical data	
			0.05% (12.5 µL)	80%	2.0 × 10^6/mL	20°C, 144h (darkness)	Graphical data	
			0.2% (50 µL)	30-40%	2.0 × 10^6/mL	20°C, 24h (darkness)	Graphical data	
			0.2% (50 µL)	40-50%	2.0 × 10^6/mL	20°C, 48h (darkness)	Graphical data	
			0.2% (50 µL)	40-50%	2.0 × 10^6/mL	20°C, 72h (darkness)	Graphical data	
			0.2% (50 µL)	50-60%	2.0 × 10^6/mL	20°C, 96h (darkness)	Graphical data	
			0.2% (50 µL)	60%	2.0 × 10^6/mL	20°C, 120h (darkness)	Graphical data	
			0.2% (50 µL)	60-70%	2.0 × 10^6/mL	20°C, 144h (darkness)	Graphical data	
Fusarium oxysporum FoCBS129	*Fucus vesiculosus*	Supercritical fluid extraction (CO2)	0.05% (12.5 µL)	30-40%	2.0 × 10^6/mL	20°C, 24h (darkness)	Graphical data	[20]
			0.05% (12.5 µL)	30-40%	2.0 × 10^6/mL	20°C, 48h (darkness)	Graphical data	
			0.05% (12.5 µL)	50-60%	2.0 × 10^6/mL	20°C, 72h (darkness)	Graphical data	
			0.05% (12.5 µL)	40-50%	2.0 × 10^6/mL	20°C, 96h (darkness)	Graphical data	
			0.05% (12.5 µL)	40-50%	2.0 × 10^6/mL	20°C, 120h (darkness)	Graphical data	
			0.05% (12.5 µL)	50-60%	2.0 × 10^6/mL	20°C, 144h (darkness)	Graphical data	
			0.2% (50 µL)	10-20%	2.0 × 10^6/mL	20°C, 24h (darkness)	Graphical data	
			0.2% (50 µL)	0-10%	2.0 × 10^6/mL	20°C, 48h (darkness)	Graphical data	
			0.2% (50 µL)	20-30%	2.0 × 10^6/mL	20°C, 72h (darkness)	Graphical data	
			0.2% (50 µL)	30-40%	2.0 × 10^6/mL	20°C, 96h (darkness)	Graphical data	
			0.2% (50 µL)	30-40%	2.0 × 10^6/mL	20°C, 120h (darkness)	Graphical data	
			0.2% (50 µL)	30-40%	2.0 × 10^6/mL	20°C, 144h (darkness)	Graphical data	
			0.05% (12.5 µL)	30-40%	2.0 × 10^6/mL	20°C, 24h (darkness)	Graphical data	
			0.05% (12.5 µL)	20-30%	2.0 × 10^6/mL	20°C, 48h (darkness)	Graphical data	
			0.05% (12.5 µL)	20-30%	2.0 × 10^6/mL	20°C, 72h (darkness)	Graphical data	
			0.05% (12.5 µL)	20-30%	2.0 × 10^6/mL	20°C, 96h (darkness)	Graphical data	
			0.05% (12.5 µL)	10-20%	2.0 × 10^6/mL	20°C, 120h (darkness)	Graphical data	
			0.05% (12.5 µL)	10-20%	2.0 × 10^6/mL	20°C, 144h (darkness)	Graphical data	
			0.2% (50 µL)	0-10%	2.0 × 10^6/mL	20°C, 48h (darkness)	Graphical data	
			0.2% (50 µL)	0-10%	2.0 × 10^6/mL	20°C, 72h (darkness)	Graphical data	
			0.2% (50 µL)	0-10%	2.0 × 10^6/mL	20°C, 96h (darkness)	Graphical data	
			0.2% (50 µL)	0-10%	2.0 × 10^6/mL	20°C, 120h (darkness)	Graphical data	
			0.2% (50 µL)	0-10%	2.0 × 10^6/mL	20°C, 144h (darkness)	Graphical data	

Table S6. Data available about the antifungal activity against phytopathogenic fungi from macroalgae using the broth microdilution assay.

Broth microdilution assay							
**Phytopathogenic Fungi**	**Macrolgae source**	**Extraction solvent**	**Extract composition**	**Extract concentration for inhibition**	**Incubation conditions**	**Notes**	**Reference**
Alternaria alternata	*Hormophysa cuneiformis*	Chloroform	Saturated fatty acids (palmitic (C16:0), myristic (C14:0) and stearic (C18:0) fatty acids)/Unsaturated fatty acids (MUFAs: oleic (C18:1, ω–9) and palmitoleic (C16:1, ω–7) acids)/PUFAs: arachidonic (C20:4, ω–6), dihomo-γ-linolenic (C20:3, ω–6) and cis-11,14-eicosadienoic (C20:2, ω–6) fatty acids); essential oils	6,25 µg/mL	30ºC, 4-7 days	Fungistatic activity	[21]
Aspergillus fumigatus	*Osmundea pinnatifida*	Dichloro-methane	-	100 µg/mL	30ºC, 4-7 days	MEC (minimum effective concentration defined as "the lowest concentration of the drug yielding morphological alterations")	[22]*C
		n-Hexane	Mainly palmitic acid (hexadecanoic acid, 29.60% ± 0.60), phytol isomer 1 (12.80% ± 0.30), oleic acid (octadecenoic acid, 9.62% ± 0.14), stearic acid (octadecanoid acid, 6.15% ± 0.02) and D-(−)-tagatofuranose (4.11% ± 0.04).	10 µg/mL	30ºC, 4-7 days		
Cladosporium herbarum	*Hormophysa cuneiformis*	Chloroform	Saturated fatty acids (palmitic (C16:0), myristic (C14:0) and stearic (C18:0) fatty acids)/Unsaturated fatty acids (MUFAs: oleic (C18:1, ω–9) and palmitoleic (C16:1, ω–7) acids)/PUFAs: arachidonic (C20:4, ω–6), dihomo-γ-linolenic (C20:3, ω–6) and cis-11,14-eicosadienoic (C20:2, ω–6) fatty acids); essential oils	1,56 µg/mL	30ºC, 4-7 days	Fungistatic activity	[21]
Fusarium oxysporum	*Hormophysa cuneiformis*	Chloroform	Saturated fatty acids (palmitic (C16:0), myristic (C14:0) and stearic (C18:0) fatty acids)/Unsaturated fatty acids (MUFAs: oleic (C18:1, ω–9) and palmitoleic (C16:1, ω–7) acids)/PUFAs: arachidonic (C20:4, ω–6), dihomo-γ-linolenic (C20:3, ω–6) and cis-11,14-eicosadienoic (C20:2, ω–6) fatty acids); essential oils	6,25 µg/mL	30ºC, 48h (orbital shaking)	Fungistatic activity	[21]
Penicillum digitatum	*Hormophysa cuneiformis*	Chloroform	Saturated fatty acids (palmitic (C16:0), myristic (C14:0) and stearic (C18:0) fatty acids)/Unsaturated fatty acids (MUFAs: oleic (C18:1, ω–9) and palmitoleic (C16:1, ω–7) acids)/PUFAs: arachidonic (C20:4, ω–6), dihomo-γ-linolenic (C20:3, ω–6) and cis-11,14-eicosadienoic (C20:2, ω–6) fatty acids); essential oils	6,25 µg/mL	30ºC, 48h (orbital shaking)	Fungistatic activity	[21]
Pseudocercospora fijiensis	*Halymenia floresii*	Aqueous solvent	-	13.7 mg/mL	27 ± 3ºC, 72 h	Fungistatic activity (positive control described as fungicide)	[23]
			-	27.3 mg/mL	28 ± 3ºC, 72 h		
		Ultrafiltration technique	Protein fraction	0.625 µg/mL	29 ± 3ºC, 72 h		
				1.25 µg/mL	30 ± 3ºC, 72 h		
				5 µg/mL	31 ± 3ºC, 72 h		
				2.5 µg/mL	32 ± 3ºC, 72 h		

*[C] - The authors predicted “that the antifungal effect of this extract is due to the presence of D-(−)-tagatofuranose, that harbours a carbohydrate ring with structural homology to the chitin synthase inhibitors nikkomycin Z, polyoxin A and polyoxin B."

Table S7. Data available about the antifungal activity against phytopathogenic fungi from macroalgae by the evaluation of inhibition of mycelial growth (by spraying the fungi culture with macroalgae extract).

Inhibition of mycelial growth								
**Phytophatogenic fungi**	**Macroalgae identification**	**Methodology of extraction/solvent used**	**Composition of the extract**	**Extract concentration**	**Inhibition of the mycelial growth**	**Incubation conditions**	**Notes**	**Reference**
Fusarium culmorum FcCBS122	*Fucus vesiculosus*	Supercritical fluid extraction (CO2)	Polyssacharides (fucosterol, fucoidans), fucoxanthin, alginic acid, laminaran, mannitol sugar alcohol and sugar polymers	1% extract (1 mL aqueous suspension)	17%	20°C, 72h	-	[20]
					10-20%	20°C, 96h	Graphical values	
					10-20%	20°C, 120h	Graphical values	
					11%	20°C, 168h	-	
				100% extrac (30 mg)	70%	20°C, 72h	-	
					80%	20°C, 96h	-	
					70-80%	20°C, 120h	Graphical values	
					70-80%	20°C, 168h	Graphical values	
Fusarium culmorum DEM Fc37	*Fucus vesiculosus*	Supercritical fluid extraction (CO2)	Polyssacharides (fucosterol, fucoidans), fucoxanthin, alginic acid, laminaran, mannitol sugar alcohol and sugar polymers	1% extract (1 mL aqueous suspension)	29%	20°C, 72h	-	
					30-40%	20°C, 96h	Graphical values	
					30-40%	20°C, 120h	Graphical values	
					39%	20°C, 168h	-	
				100% extract (30 mg)	53%	20°C, 72h	-	
					40-50%	20°C, 96h	Graphical values	
					40-50%	20°C, 120h	Graphical values	
					30-40%	20°C, 168h	Graphical values	
Fusarium oxysporum FoCBS129	*Fucus vesiculosus*	Supercritical fluid extraction (CO2)	Polyssacharides (fucosterol, fucoidans), fucoxanthin, alginic acid, laminaran, mannitol sugar alcohol and sugar polymers	1% extract (1 mL aqueous suspension)	28%	20°C, 72h	-	
					20-30%	20°C, 96h	Graphical values	
					20-30%	20°C, 120h	Graphical values	
					16%	20°C, 168h	-	
				100% extrac (30 mg)	57%	20°C, 72h	-	
					40-50%	20°C, 96h	Graphical values	
					50-60%	20°C, 120h	Graphical values	
					50-60%	20°C, 168h	Graphical values	
Fusarium oxysporum DEM Fo38	*Fucus vesiculosus*	Supercritical fluid extraction (CO2)	Polyssacharides (fucosterol, fucoidans), fucoxanthin, alginic acid, laminaran, mannitol sugar alcohol and sugar polymers	1% extract (1 mL aqueous suspension)	40%	20°C, 72h	-	
					40%	20°C, 96h	Graphical values	
					30-40%	20°C, 120h	Graphical values	
					48%	20°C, 168h	-	
				100% extract (30 mg)	60%	20°C, 72h	-	
					60-70%	20°C, 96h	Graphical values	
					50-60%	20°C, 120h	Graphical values	
					75%	20°C, 168h	-	

Table S8. Data available about the antifungal activity against phytopathogenic fungi from macroalgae by the evaluation of fungal spore germination.

Fungal Spore Germination								
**Phytopathogenic fungi**	**Macroalgae source**	**Solvent used**	**Compounds proposed (bibliographic information)**	**Antifungal compound proposed**	**Extract concentration (%)**	**Germination (%)**	**Initial spore concentration**	**Reference**
Rhizoctonia solani Kuhn	*Dictyota dichotoma*	Chloroform:methanol 1:1 (v/v)	-	-	5%	35,50%	1*10^6 spore/mL	[12]
			-	-	10%	33,80%		
			-	-	15%	25,50%		
			-	-	20%	17,20%		
	Padina gymnospora	Chloroform:methanol 1:1 (v/v)	-	-	5%	37,80%	1*10^6 spore/mL	
			-	-	10%	34,23%		
			-	-	15%	32,11%		
			-	-	20%	20,10%		
	Sargassum muticum	Chloroform:methanol 1:1 (v/v)	-	-	5%	39,80%	1*10^6 spore/mL	
			-	-	10%	36,71%		
			-	-	15%	30,86%		
			-	-	20%	24,30%		
	Sargassum tenerrimum	Chloroform:methanol 1:1 (v/v)	n-hexadecanoic acid; Geranyl isovalerate; Oleic acid; 17-Octadecynoic acid; 9,12,15-Octadecatrienoic acid, 2,3-dihydroxypropyl ester, (Z,Z,Z)-; trans-13-Octadecenoic acid; 11-Octadecenoic acid, methyl ester; 6,9,12,15-Docosatetraenoic acid, methyl ester; Gibberellic acid; Fenretinide; 9,10-Secocholesta-5,7,10(19)-triene-3,24,25-triol, (3β,5Z,7E)-; Cholestan-3-ol, 2-methylene-, (3β,5α)-	n-hexadecanoid acid	5%	45,70%	1*10^6 spore/mL	
					10%	40,30%		
					15%	36,20%		
					20%	28,80%		
	Sargassum wightii	Chloroform:methanol 1:1 (v/v)	-	-	5%	42,50%	1*10^6 spore/mL	
			-	-	10%	39,41%		
			-	-	15%	28,77%		
			-	-	20%	26,61%		

Table S9. Data available about the antifungal activity against phytopathogenic fungi from macroalgae by the spore spreading method.

Spore Spreading Method								
**Phytopathogenic fungi**	**Macroalgae source**	**Solvent used**	**Compounds proposed (bibliographic information)**	**Extract concentraction**	**Halo inhibition (mm)**	**Medium**	**Incubation**	**Reference**
Pyricularia oryzae	*Rhodomela confervoides*	Methanol	Brominated compounds	30 µL/disc	20.0±1.0 mm	Sabouraud's medium	30°C, 24-48h	[24]
	Symphyocladia latiuscula	Methanol	Brominated compounds		35.0±2.0 mm			

Note: Procedure detailed by Zhang [24].

Table S10. Data available about the antifungal activity against phytopathogenic fungi from macroalgae by the fungal germination in test tube.

Germination in test tube (supplemented with agar)							
**Phytopathogenic fungi**	**Macroalgae source**	**Solvent used**	**Extract concentration (µg/mL)**	**Inhibition of growth**	**Medium**	**Incubation**	**Reference**
Fusarium moniliforme	*Botryocladia leptopoda*	Methanolic extract	400 µg/mL	66,6%	Sabouraud Dextrose agar	27-29°C, 7-10 days	[25]
	Dictyota hauckiana	Methanolic extract	400 µg/mL	50%	Sabouraud Dextrose agar	27-29°C, 7-10 days	
Fusarium solani	*Botryocladia leptopoda*	Methanolic extract	400 µg/mL	60%	Sabouraud Dextrose agar	27-29°C, 7-10 days	
	Caulerpa racemosa	Methanolic extract	400 µg/mL	50%	Sabouraud Dextrose agar	27-29°C, 7-10 days	
	Caulerpa taxifolia	Methanolic extract	400 µg/mL	55%	Sabouraud Dextrose agar	27-29°C, 7-10 days	
	Champia compressa	Methanolic extract	400 µg/mL	60%	Sabouraud Dextrose agar	27-29°C, 7-10 days	
	Codium indicum	Methanolic extract	400 µg/mL	56,3%	Sabouraud Dextrose agar	27-29°C, 7-10 days	
	Gracilaria corticata	Methanolic extract	400 µg/mL	14,2%	Sabouraud Dextrose agar	27-29°C, 7-10 days	
	Hypnea musciformis	Methanolic extract	400 µg/mL	70%	Sabouraud Dextrose agar	27-29°C, 7-10 days	
	Hypnea valentiae	Methanolic extract	400 µg/mL	58,3%	Sabouraud Dextrose agar	27-29°C, 7-10 days	
	Osmundea pinnatifida	Methanolic extract	400 µg/mL	28,57%	Sabouraud Dextrose agar	27-29°C, 7-10 days	
	Padina antillarum	Methanolic extract	400 µg/mL	51.6%	Sabouraud Dextrose agar	27-29°C, 7-10 days	
	Sarconema filiforme	Methanolic extract	400 µg/mL	60%	Sabouraud Dextrose agar	27-29°C, 7-10 days	
	Sargassum ilicifolium	Methanolic extract	400 µg/mL	44.28%	Sabouraud Dextrose agar	27-29°C, 7-10 days	
	Sargassum vulgare	Methanolic extract	400 µg/mL	4.2%	Sabouraud Dextrose agar	27-29°C, 7-10 days	
	Solieria robusta	Methanolic extract	400 µg/mL	40%	Sabouraud Dextrose agar	27-29°C, 7-10 days	
	Stoechospermum polypodioides	Methanolic extract	400 µg/mL	58.3%	Sabouraud Dextrose agar	27-29°C, 7-10 days	
	Ulva lactuca	Methanolic extract	400 µg/mL	55,71%	Sabouraud Dextrose agar	27-29°C, 7-10 days	
	Ulva lactuca	Methanolic extract	400 µg/mL	4,2%	Sabouraud Dextrose agar	27-29°C, 7-10 days	
Mucor sp.	*Champia compressa*	Methanolic extract	400 µg/mL	2.66%	Sabouraud Dextrose agar	27-29°C, 7-10 days	
	Hypnea musciformis	Methanolic extract	400 µg/mL	6.66%	Sabouraud Dextrose agar	27-29°C, 7-10 days	
	Sargassum boveanum	Methanolic extract	400 µg/mL	8.00%	Sabouraud Dextrose agar	27-29°C, 7-10 days	
	Sargassum ilicifolium	Methanolic extract	400 µg/mL	4.00%	Sabouraud Dextrose agar	27-29°C, 7-10 days	
	Ulva lactuca	Methanolic extract	400 µg/mL	20%	Sabouraud Dextrose agar	27-29°C, 7-10 days	

Table S11. Data available about the antifungal activity against phytopathogenic fungi from macroalgae tested in field studies.

Field studies												
**Phytopathogenic fungi**	**Macroalgae source**	**Type of the extract applied/ concentration**	**First extract application**			**Second extract application**			**Host**	**Notes**	**Reference**
			**Infection after application (%)**	**Control infection (%)**	**Collection after infection (days)**	**Infection after application (%)**	**Control infection (%)**	**Collection after infection (days)**			
Fusarium oxysporum	*Dictyota cervicornis*	Dry powder	6.2	43.7	30	6.2	18.7	60	*Glycine max* (L.)	-	[26]
	Halimeda tuna	Dry powder	6.2	43.7	30	0	18.7	60	*Glycine max* (L.)	-	[26]
	Melanothamnus afaqhusainii	Dry powder	0	6.2	7 weeks	-	-	-	Eggplant (*Solanum melongena* L.)	-	[27]
		Dry powder	0	18.7	7 weeks	-	-	-	Watermelon (*Citrullus lanatus* (Thunb.) Matsum. & Nakai)	-	[27]
		Dry powder	0	0	7 weeks	-	-	-	Watermelon (*Citrullus lanatus* (Thunb.) Matsum. & Nakai)	-	[27]
	Padina tetrastromatica	Dry powder	0	43.7	30	12.5	18.7	60	*Glycine max* (L.)	-	[26]
	Polycladia indica	Dry powder	0	6.2	7 weeks	-	-	-	Eggplant (*Solanum melongena* L.)	-	[27]
		Dry powder	0	18.7	7 weeks	-	-	-	Watermelon (*Citrullus lanatus* (Thunb.) Matsum. & Nakai)	-	[27]
		Dry powder	0	0	7 weeks	-	-	-	Watermelon (*Citrullus lanatus* (Thunb.) Matsum. & Nakai)	-	[27]
		Dry powder	25	43.7	30	6.2	18.7	60	*Glycine max* (L.)	-	[26]
	Sargassum swartzii	Dry powder	18.7	43.7	30	12.5	18.7	60	*Glycine max* (L.)	-	[26]
	Solieria robusta	Dry powder	0	43.7	30	0	18.7	60	*Glycine max* (L.)	-	[26]
	Spatoglossum variabile	Dry powder	0	6.2	7 weeks	-	-	-	Eggplant (*Solanum melongena* L.)	-	[27]
		Dry powder	6.2	18.7	7 weeks	-	-	-	Watermelon (*Citrullus lanatus* (Thunb.) Matsum. & Nakai)	-	[27]
		Dry powder	0	0	7 weeks	-	-	-	Watermelon (*Citrullus lanatus* (Thunb.) Matsum. & Nakai)	-	[27]
	Stoechospermum polypodioides	Dry powder	0	43.7	30	0	18.7	60	*Glycine max* (L.)	-	[26]
Fusarium solani	*Dictyota cervicornis*	Dry powder	0	25	30	18.7	75	60	*Glycine max* (L.)	-	[26]

**Field studies**											
	Halimeda tuna	Dry powder	18.7	25	30	12.5	75	60	*Glycine max* (L.)	-	[26]
		Dry powder	25	62.5	45	0	31.2	90	*Capsicum annuum* L.	-	[26]
		Dry powder	0	25	-	-	-	-	*Gossypium hirsutum* L.	-	[28]
		Dry powder	0	12,50	-	-	-	-	Sun flower	The antimicrobial activity can be due the presence of acrylic acid	[29]
		Dry powder	25	43,70	-	-	-	-	*Lycopersicum esculentum*	The antimicrobial activity can be due the presence of acrylic acid	[29]
	Melanothamnus afaqhusainii	Dry powder	25	31.2	7 weeks	-	-	-	Eggplant (*Solanum melongena* L.)		[27]
		Dry powder	31.2	37.5	7 weeks	-	-	-	Watermelon (*Citrullus lanatus* (Thunb.) Matsum. & Nakai)	Farmer´s field experiment	[27]
		Dry powder	0	43,70	-	-	-	-	*Lycopersicum esculentum*	The antimicrobial activity can be due the presence of acrylic acid	[29]
	Padina tetrastromatica	Dry powder	0	25	30	56.2	75	60	*Glycine max* (L.)	-	[26]
		Dry powder	25	62.5	45	0	31.2	90	*Capsicum annuum* L.	-	[26]
	Polycladia indica	Dry powder	18.7	37.5	7 weeks				Watermelon (*Citrullus lanatus* (Thunb.) Matsum. & Nakai)	-	[27]
		Dry powder	12.5	25	30	25	75	60	*Glycine max* (L.)	-	[26]
		Dry powder	6.2	62.5	45	6.2	31.2	90	*Capsicum annuum* L.	-	[26]
		Dry powder	12,5	25	-	-	-	-	*Gossypium hirsutum* L.	-	[28]
	Rhizoclonium riparium	Dry powder	18,7	25	-	-	-	-	*Gossypium hirsutum* L.	-	[28]
	Sargassum aquifolium	Dry powder	18.7	62.5	45	6.2	31.2	90	*Capsicum annuum* L.	-	[26]
	Sargassum swartzii	Dry powder	-	-	-	-	-	-	*Lycopersicum esculentum*	Effective	[30]
		Dry powder	-	-	-	-	-	-	*Lycopersicum esculentum*	Effective	[30]
		Dry powder	0	25	30	43.7	75	60	*Glycine max* (L.)	-	[26]
	Sargassum tenerrimum	Dry powder	18.7	62.5	45	6.2	31.2	90	*Capsicum annuum* L.	-	[26]
	Solieria robusta	Powder	-	-	-	-	-	-		-	[31]
		Dry powder	6.2	25	30	31.2	75	60	*Glycine max* (L.)	-	[26]
		Dry powder	18.7	62.5	45	0	31.2	90	*Capsicum annuum* L.	-	[26]
		Dry powder	18,7	25	-				*Gossypium hirsutum* L.	-	[28]

**Field studies**											
	Spatoglossum asperum	Dry powder	-	-	-	-	-	-	*Lycopersicum esculentum*	Effective	[30]
		Dry powder	-	-	-	-	-	-	*Lycopersicum esculentum*	Effective	[30]
		Dry powder	18.7	31.2	7 weeks	-	-	-	Eggplant (*Solanum melongena* L.)	-	[27]
		Dry powder	31.2	37.5	7 weeks	-	-	-	Watermelon (*Citrullus lanatus* (Thunb.) Matsum. & Nakai)	-	[27]
	Spatoglossum variabile	Dry powder	18.7	37.5	7 weeks	-	-	-	Watermelon (*Citrullus lanatus* (Thunb.) Matsum. & Nakai)	Farmer´s field experiment	[27]
		-	0	12,50	-	-	-	-	Sun flower	The antimicrobial activity can be due the presence of acrylic acid	[29]
		-	0	43,70	-	-	-	-	*Lycopersicum esculentum*	The antimicrobial activity can be due the presence of acrylic acid	[29]
	Stoechospermum polypodioides	Dry powder	0	25	30	31.2	75	60	*Glycine max* (L.)	-	[26]
		Dry powder	6.2	62.5	45	0	31.2	90	*Capsicum annuum* L.	-	[26]
Macrophomina phaseolina	*Dictyota cervicornis*	Dry powder	0	0	30	6.2	25	60	*Glycine max* (L.)	-	[26]
	Halimeda tuna	Dry powder	0	0	30	0	25	60	*Glycine max* (L.)	-	[26]
		Dry powder	0	45	45	12.5	25	90	*Capsicum annuum* L.	-	[26]
		-	0	18,70	-	-	-	-	*Gossypium hirsutum* L.	The antimicrobial activity can be due the presence of acrylic acid.	[29]
		-	0	25	-	-	-	-	Sun flower	-	[28]
	Melanothamnus afaqhusainii	Dry powder	12.5	68.7	7 weeks	-	-	-	Eggplant (*Solanum melongena* L.)	-	[27]
		Dry powder	0	31.2	7 weeks	-	-	-	Watermelon (*Citrullus lanatus* (Thunb.) Matsum. & Nakai)	-	[27]
		Dry powder	0	18.7	7 weeks	-	-	-	Watermelon (*Citrullus lanatus* (Thunb.) Matsum. & Nakai)	Farmer´s field experiment	[27]
		Dry powder	6,2	25	-	-	-	-	*Gossypium hirsutum* L.	-	[28]
		Dry powder	0	6,20	-	-	-	-	*Lycopersicum esculentum*	The antimicrobial activity can be due the presence of acrylic acid.	[29]

**Field studies**											
	Padina tetrastromatica	Dry powder	0	45	45	0	25	90	*Capsicum annuum* L.	-	[26]
	Polycladia indica	Dry powder	0	68.7	7 weeks	-	-	-	Eggplant (*Solanum melongena* L.)	-	[27]
		Dry powder	0	18.7	7 weeks	-	-	-	Watermelon (*Citrullus lanatus* (Thunb.) Matsum. & Nakai)	Farmer´s field experiment	[27]
		Dry powder	0	0	30	12.5	25	60	*Glycine max* (L.)	-	[26]
		Dry powder	0	45	45	6.2	25	90	*Capsicum annuum* L.	-	[26]
		Dry powder	6,2	25	-	-	-	-	*Gossypium hirsutum* L.	-	[28]
	Rhizoclonium riparium	Dry powder	12,5	25	-	-	-	-	*Gossypium hirsutum* L.	-	[28]
	Sargassum aquifolium	Dry powder	0	45	45	6.2	25	90	*Capsicum annuum* L.	-	[26]
		Dry powder	12,5	25	-	-	-	-	*Gossypium hirsutum* L.	-	[28]
	Sargassum swartzii	0.5%	-	-	-	-	-	-	*Lycopersicum esculentum*	Complete suppresion	[30]
		1%	-	-	-	-	-	-	*Lycopersicum esculentum*	Complete suppresion	[30]
	Sargassum tenerrimum	Dry powder	0	45	45	6.2	25	90	*Capsicum annuum* L.	-	[26]
	Solieria robusta	Dry powder	-	-	-	-	-	-		-	[31]
		Dry powder	0	0	30	0	25	60	*Glycine max* (L.)	-	[26]
		Dry powder	6.2	45	45	0	25	90	*Capsicum annuum* L.	-	[26]
		Dry powder	0	25	-	-	-	-	*Gossypium hirsutum* L.	-	[28]
	Spatoglossum asperum	0.5%	-	-	-	-	-	-	*Lycopersicum esculentum*	Effective	[30]
		1%	-	-	-	-	-	-	*Lycopersicum esculentum*	Complete suppresion	[30]
	Spatoglossum variabile	Dry powder	0	68.7	7 weeks	-	-	-	Eggplant (*Solanum melongena* L.)	-	[27]
		Dry powder	6.2	31.2	7 weeks	-	-	-	Watermelon (*Citrullus lanatus* (Thunb.) Matsum. & Nakai)	-	[27]
		Dry powder	0	18.7	7 weeks	-	-	-	Watermelon (*Citrullus lanatus* (Thunb.) Matsum. & Nakai)	Farmer´s field experiment	[27]
		Dry powder	6,2	25	-	-	-	-	*Gossypium hirsutum* L.	-	[28]
		Dry powder	0	18,70	-	-	-	-	Sun flower	The antimicrobial activity can be due the presence of acrylic acid	[29]
		Dry powder	0	6,20	-	-	-	-	*Lycopersicum esculentum*	The antimicrobial activity can be	[29]

**Field studies**											
										due the presence of acrylic acid	
	Stoechospermum polypodioides	Dry powder	0	0	30	12.5	25	60	*Glycine max* (L.)	-	[26]
		Dry powder	0	45	45	0	25	90	*Capsicum annuum* L.	-	[26]
		Dry powder	0	25	-	-	-	-	*Gossypium hirsutum* L.	-	[28]
Rhizoctonia solani	*Dictyota cervicornis*	Dry powder	6.2	62.5	30	62.5	87.5	60	*Glycine max* (L.)	-	[26]
	Halimeda tuna	Dry powder	18.7	62.5	30	50	87.5	60	*Glycine max* (L.)	-	[26]
	Halimeda tuna	Dry powder	0	31.2	45	6.2	25	90	*Capsicum annuum* L.	-	[26]
		Dry powder	-	31.2	-	-	-	-	*Gossypium hirsutum* L.	-	[28]
		Dry powder	0	12,50	-	-	-	-	Sun flower	The antimicrobial activity can be due the presence of acrylic acid.	[29]
		Dry powder	12,5	56,20	-	-	-	-	*Lycopersicum esculentum*	The antimicrobial activity can be due the presence of acrylic acid.	[29]
	Melanothamnus afaqhusainii	Dry powder	0	25	7 weeks	-	-	-	Watermelon (*Citrullus lanatus* (Thunb.) Matsum. & Nakai)	-	[27]
		Dry powder	0	6.2	7 weeks	-	-	-	Watermelon (*Citrullus lanatus* (Thunb.) Matsum. & Nakai)	Farmer´s field experiment	[27]
		Dry powder	18,7	31.2	-	-	-	-	*Gossypium hirsutum* L.	-	[28]
		Dry powder	12,5	56,20	-	-	-	-	*Lycopersicum esculentum*	The antimicrobial activity can be due the presence of acrylic acid.	[29]
	Padina tetrastromatica	Dry powder	12.5	62.5	30	56.2	87.5	60	*Glycine max* (L.)	-	[26]
		Dry powder	0	31.2	45	0	25	90	*Capsicum annuum* L.	-	[26]
	Polycladia indica	Dry powder	0	6.2	7 weeks	-	-	-	Eggplant (*Solanum melongena* L.)	-	[27]
		Dry powder	12.5	25	7 weeks	-	-	-	Watermelon (*Citrullus lanatus* (Thunb.) Matsum. & Nakai)	-	[27]
		Dry powder	31.2	62.5	30	56.2	87.5	60	*Glycine max* (L.)	-	[26]
		Dry powder	0	31.2	45	0	25	90	*Capsicum annuum* L.	-	[26]
		Dry powder	6,2	31.2	-	-	-	-	*Gossypium hirsutum* L.	-	[28]
	Rhizoclonium riparium	Dry powder	25	31.2	-	-	-	-	*Gossypium hirsutum* L.	-	[28]

**Field studies**											
	Sargassum aquifolium	Dry powder	0	31.2	45	0	25	90	*Capsicum annuum* L.	-	[26]
		Dry powder	18,7	31.2	-	-	-	-	*Gossypium hirsutum* L.	-	[28]
	Sargassum swartzii	Dry powder	-	-	-	-	-	-	*Lycopersicum esculentum*	Complete suppresion	[30]
		Dry powder	-	-	-	-	-	-	*Lycopersicum esculentum*	Complete suppresion	[30]
		Dry powder	25	62.5	30	50	87.5	60	*Glycine max* (L.)	-	[26]
	Sargassum tenerrimum	Dry powder	0	31.2	45	6.2	25	90	*Capsicum annuum* L.	-	[26]
	Solieria robusta	Dry powder	-	-	-	-	-	-		-	[31]
		Dry powder	18.7	62.5	30	25	87.5	60	*Glycine max* (L.)	-	[26]
		Dry powder	0	31.2	45	0	25	90	*Capsicum annuum* L.	-	[26]
		Dry powder	0	31.2	-	-	-	-	*Gossypium hirsutum* L.	-	[28]
	Spatoglossum asperum	Dry powder	-	-	-	-	-	-	*Lycopersicum esculentum*	Effective	[30]
		Dry powder	-	-	-	-	-	-	*Lycopersicum esculentum*	Effective	[30]
	Spatoglossum variabile	Dry powder	6.2	25	7 weeks	-	-	-	Watermelon (*Citrullus lanatus* (Thunb.) Matsum. & Nakai)	-	[27]
		Dry powder	0	6.2	7 weeks	-	-	-	Watermelon (*Citrullus lanatus* (Thunb.) Matsum. & Nakai)	Farmer´s field experiment	[27]
		Dry powder	25	31.2	-	-	-	-	*Gossypium hirsutum* L.	-	[28]
		Dry powder	12,5	56,20	-	-	-	-	*Lycopersicum esculentum*	The antimicrobial activity can be due the presence of acrylic acid.	[29]
	Stoechospermum polypodioides	Dry powder	18.7	62.5	30	18.7	87.5	60	*Glycine max* (L.)	-	[26]
		Dry powder	0	31.2	45	0	25	90	*Capsicum annuum* L.	-	[26]
		Dry powder	18,7	31.2	-	-	-	-	*Gossypium hirsutum* L.		[28]

Table S12. Data available about the antifungal activity against phytopathogenic fungi from macroalgae tested in screenhouse studies.

Screenhouse studies (analysed of the infection using pots)							
**Phytopathogenic fungi**	**Macroalgae source**	**Type of the extract applied/ concentration**	**Infection after tretment (%)**	**Infection in the control (%)**	**Host**	**Notes**	**Reference**
Fusarium solani	*Dictyota cervicornis*	Dry powder	6.2	25	*Glycine max* (L.)	-	[26]
	Halimeda tuna	Dry powder	12.5	25	*Glycine max* (L.)	-	[26]
		Dry powder	12,5	31.2	*Gossypium hirsutum* L.	-	[28]
		Dry powder	25	43,70	Sun flower	The antimicrobial activity can be due the presence of acrylic acid	[29]
		Dry powder	12,5	37,50	*Lycopersicum esculentum*	The antimicrobial activity can be due the presence of acrylic acid	[29]
	Melanothamnus afaqhusainii	Dry powder	25	31.2	*Gossypium hirsutum* L.	-	[28]
		Dry powder	25	43,70	Sun flower	The antimicrobial activity can be due the presence of acrylic acid	[29]
	Padina tetrastromatica	Dry powder	12.5	25	*Glycine max* (L.)	-	[26]
	Polycladia indica	Dry powder	18.7	25	*Glycine max* (L.)	-	[26]
		Dry powder	6,2	31.2	*Gossypium hirsutum* L.	-	[28]
	Rhizoclonium riparium	Dry powder	18,7	31.2	*Gossypium hirsutum* L.	-	[28]
	Sargassum aquifolium	Dry powder	6,2	31.2	*Gossypium hirsutum* L.	-	[28]
	Sargassum swartzii	Dry powder (0.5%)	31,2	81,5	*Lycopersicum esculentum*	Effective	[30]
		Dry powder (1%)	12,5	81,5	*Lycopersicum esculentum*	Effective	[30]
		Dry powder	6.2	25	*Glycine max* (L.)	-	[26]
	Solieria robusta	Dry powder	0	25	*Glycine max* (L.)	-	[26]
		Dry powder	12,5	31.2	*Gossypium hirsutum* L.	-	[28]
	Spatoglossum asperum	Dry powder (0.5%)	43,7	75	*Lycopersicum esculentum*	Effective	[30]
		Dry powder (1%)	43,7	75	*Lycopersicum esculentum*	Effective	[30]
	Spatoglossum variabile	Dry powder	18,7	31.2	*Gossypium hirsutum* L.	-	[28]
		Dry powder	18,7	43,70	Sun flower	The antimicrobial activity can be due the presence of acrylic acid	[29]
		Dry powder	12,5	37,50	*Lycopersicum esculentum*	The antimicrobial activity can be due the presence of acrylic acid	[29]
	Stoechospermum polypodioides	Dry powder	12.5	25	*Glycine max* (L.)	-	[26]
		Dry powder	18,7	31.2	*Gossypium hirsutum* L.	-	[28]
Macrophomina phaseolina	*Dictyota cervicornis*	Dry powder	6.2	18.7	*Glycine max* (L.)	-	[26]
	Halimeda tuna	Dry powder	0	18.7	*Glycine max* (L.)	-	[26]

Screenhouse studies (analysed of the infection using pots)							
		Dry powder	18,7	43.7	*Gossypium hirsutum* L.	-	[28]
		Dry powder	12,5	31.2	Sun flower	The antimicrobial activity can be due the presence of acrylic acid.	[29]
	Melanothamnus afaqhusainii	Dry powder	12,5	43.7	*Gossypium hirsutum* L.	-	[28]
		Dry powder	18,7	31.2	Sun flower	The antimicrobial activity can be due the presence of acrylic acid.	[29]
		Dry powder	0	6,20	*Lycopersicum esculentum*	The antimicrobial activity can be due the presence of acrylic acid.	[29]
	Polycladia indica	Dry powder	0	18.7	*Glycine max* (L.)	-	[26]
		Dry powder	25	43.7	*Gossypium hirsutum* L.	-	[28]
	Rhizoclonium riparium	Dry powder	6,2	43.7	*Gossypium hirsutum* L.	-	[28]
	Sargassum aquifolium	Dry powder	12,5	43.7	*Gossypium hirsutum* L.	-	[28]
	Sargassum swartzii	Dry powder (0.5%)	0	25	*Lycopersicum esculentum*	Complete suppresion	[30]
		Dry powder (1%)	0	25	*Lycopersicum esculentum*	Complete suppresion	[30]
	Solieria robusta	Dry powder	0	18.7	*Glycine max* (L.)	-	[26]
		Dry powder	18,7	43.7	*Gossypium hirsutum* L.	-	[28]
	Spatoglossum asperum	Dry powder (0.5%)	6,2 (no units)	25 (no)	*Lycopersicum esculentum*	Effective	[30]
		Dry powder (1%)	0	25	*Lycopersicum esculentum*	Complete suppresion	[30]
	Spatoglossum variabile	Dry powder	6,2	43.7	*Gossypium hirsutum* L.	-	[28]
		Dry powder	0	31.2	Sun flower	The antimicrobial activity can be due the presence of acrylic acid.	[29]
		Dry powder	0	6,20	*Lycopersicum esculentum*	The antimicrobial activity can be due the presence of acrylic acid.	[29]
	Stoechospermum polypodioides	Dry powder	6.2	18.7	*Glycine max* (L.)	-	[26]
		Dry powder	31,2	43.7	*Gossypium hirsutum* L.	-	[28]
Rhizoctonia solani	*Dictyota cervicornis*	Dry powder	0	6.2	*Glycine max* (L.)	-	[26]
	Halimeda tuna	Dry powder	0	6.2	*Glycine max* (L.)	-	[26]
		Dry powder	12,5	37.5	*Gossypium hirsutum* L.	-	[28]
		Dry powder	6,2	25	*Lycopersicum esculentum*	The antimicrobial activity can be due the presence of acrylic acid.	[29]

Screenhouse studies (analysed of the infection using pots)							
	Melanothamnus afaqhusainii	Dry powder	18,7	37.5	*Gossypium hirsutum* L.	-	[28]
		Dry powder	18,7	25	Sun flower	The antimicrobial activity can be due the presence of acrylic acid.	[29]

		Dry powder	6,2	25	*Lycopersicum esculentum*	The antimicrobial activity can be due the presence of acrylic acid.	[29]
	Padina tetrastromatica	Dry powder	0	6.2	*Glycine max* (L.)	-	[26]
	Polycladia indica	Dry powder	0	6.2	*Glycine max* (L.)	-	[26]
		Dry powder	12,5	37.5	*Gossypium hirsutum* L.	-	[28]
	Rhizoclonium riparium	Dry powder	25	37.5	*Gossypium hirsutum* L.	-	[28]
	Sargassum aquifolium	Dry powder	6,2	37.5	*Gossypium hirsutum* L.	-	[28]
	Sargassum swartzii	Dry powder (0.5%)	0	56,2	*Lycopersicum esculentum*	Complete suppresion	[30]
		Dry powder (1%)	0	56,2	*Lycopersicum esculentum*	Complete suppresion	[30]
		Dry powder	0	6.2	*Glycine max* (L.)	-	[26]
	Solieria robusta	Dry powder	0	6.2	*Glycine max* (L.)	-	[26]
		Dry powder	12,5	37.5	*Gossypium hirsutum* L.	-	[28]
	Spatoglossum asperum	Dry powder (0.5%)	25	56,2	*Lycopersicum esculentum*	Effective	[30]
		Dry powder (1%)	6,2	56,2	*Lycopersicum esculentum*	Effective	[30]
	Spatoglossum variabile	Dry powder	18,7	37.5	*Gossypium hirsutum* L.	-	[28]
		Dry powder	12,5	25	Sun flower	The antimicrobial activity can be due the presence of acrylic acid.	[29]
		Dry powder	6,2	25	*Lycopersicum esculentum*	The antimicrobial activity can be due the presence of acrylic acid.	[29]
	Stoechospermum polypodioides	Dry powder	0	6.2	*Glycine max* (L.)	-	[26]
		Dry powder	25	37.5	*Gossypium hirsutum* L.	-	[28]

All the data information were retrieved from SCOPUS database using the following search: “(Antifung* OR fungicid*) AND (Plant* OR crop* OR agricultur* OR veget* OR phytopatho*) AND (Macroalga* OR seaweed)”

References

[1] Sivagnanam, S. P.; Yin, S.; Choi, J. H.; Park, Y. B.; Woo, H. C.; Chun, B. S. Biological Properties of Fucoxanthin in Oil Recovered from Two Brown Seaweeds Using Supercritical CO2 Extraction. *Mar. Drugs*, **2015**, *13*, 3422–3442. https://doi.org/10.3390/md13063422.

[2] Manilal, A.; Sujith, S.; Kiran, G. S.; Selvin, J.; Shakir, C.; Gandhimathi, R.; Lipton, A. P. Antimicrobial Potential and Seasonality of Red Algae Collected from the Southwest Coast of India Tested against Shrimp, Human and Phytopathogens. *Ann. Microbiol.*, **2009**, *59* (2), 207–219.

[3] Achary, A.; Muthalagu, K.; Guru, M. S. Identification of Phytochemicals from *Sargassum Wightii* against *Aedes Aegypti*. *Int. J. Pharm. Sci. Rev. Res.*, **2014**, *29* (1), 314–319.

[4] Malini, M.; Ponnanikajamideen, M.; Malarkodi, C.; Rajeshkumar, S. Explore the Antimicrobial Potential from Organic Solvents Extract of Brown Seaweed (*Sargassum Longifolium*) Alleviating to Pharmaceuticals. *Int. J. Pharm. Res.*, **2014**, *6* (1), 28–35.

[5] Belattmania, Z.; Reani, A.; Barakate, M.; Zrid, R.; Elatouani, S.; Hassouani, M.; Eddaoui, A.; Bentiss, F.; Sabour, B. Antimicrobial, Antioxidant and Alginate Potentials of *Dictyopteris Polypodioides* (Dictyotales, Phaeophyceae) from the Moroccan Atlantic Coast. *Der Pharma Chem.*, **2016**, *8* (2), 216–226.

[6] Moreau, J.; Pesando, D.; Bernard, P.; Caram, B.; Pionnat, J. C. Seasonal Variations in the Production of Antifungal Substances by Some Dictyotales (Brown Algae) from the French Mediterranean Coast. *Hydrobiologia*, **1988**, *162*, 157–162.

[7] Machado, L. P.; Matsumoto, S. T.; Jamal, C. M.; Silva, M. B.; Centeno, D. da C.; Neto, P. C.; Carvalho, L. R.; Yokoya, N. S. Chemical Analysis and Toxicity of Seaweed Extracts with Inhibitory Activity against Tropical Fruit Anthracnose Fungi. *J Sci Food Agric*, **2013**, *94*, 1739–1744. https://doi.org/10.1002/jsfa.6483.

[8] El-sheekh, M. M.; Mousa, A. S. H.; Farghl, A. A. M. Biological Control of Fusarium Wilt Disease of Tomato Plants Using Seaweed Extracts. *Arab. J. Sci. Eng.*, **2020**, *45* (6), 4557–4570. https://doi.org/10.1007/s13369-020-04518-2.

[9] Ambreen, A.; Khan, H.; Tariq, A.; Ruqqia, A.; Sultana, V.; Ara, J. Evaluation of Biochemical Component and Antimicrobial Activity of Some Seaweeeds Occurring at Karachi Coast. *Pakistan J. Bot.*, **2012**, *44* (5), 1799–1803.

[10] Ara, J.; Sultana, V.; Qasim, R.; Ehteshamul-haque, S.; Ahmad, V. U. Biological Activity of *Spatoglossum Asperum*: A Brown Alga. *Phyther. Res.*, **2005**, *19*, 618–623.

[11] Zouaoui, B.; Ghalem, B. R. The Phenolic Contents and Antimicrobial Activities of Some Marine Algae from the Mediterranean Sea (Algeria). *Russ. J. Mar. Biol.*, **2017**, *43* (6), 491–495. https://doi.org/10.1134/S1063074017060128.

[12] Graff, K. H.; Raj, T. S. Effect of Sargassum Tenerrimum on Controlling Sheath Blight of Rice Caused by *Rhizoctonia Solani* Kuhn. *Plant Arch.*, **2019**, *19*, 1132–1135.

[13] Vehapi, M.; Koçer, A. T.; Yılmaz, A.; Özçimen, D. Investigation of the Antifungal Effects of Algal Extracts on Apple-infecting Fungi. *Arch. Microbiol.*, **2020**, *202*, 455–471.

[14] Ambika, S.; Sujatha, K. Antifungal Activity of Aqueous and Ethanol Extracts of Seaweeds against Sugarcane Red Rot Pathogen (*Colletotrichum Falcatum*). *Sci. Res. Essays*, **2015**, *10* (6), 232–235. https://doi.org/10.5897/SRE2015.6198.

[15] Machado, L. P.; Matsumoto, S. T.; Cuzzuol, G. R. F.; Jr, L. F. G. O. Influence of Laboratory Cultivation on Species of Rhodophyta Physiological Evaluations and Antifungal Activity against Phytopathogens. *Rev. Ciência Agronômica*, **2014**, *45* (1), 52–61.

[16] Mani, S. D.; Nagarathnam, R. Sulfated Polysaccharide from *Kappaphycus Alvarezii* (Doty) Doty Ex P.C. Silva Primes Defense Responses against Anthracnose Disease of *Capsicum Annuum* Linn. *Algal Res.*, **2018**, *32*, 121–130. https://doi.org/10.1016/j.algal.2018.02.025.

[17] Khan, S. A.; Abid, M.; Hussain, F. Antifungal Activity of Aqueous and Methanolic Extracts of Some Seaweeds against Common Soil-Borne Plant Pathogenic Fungi. *Pakistan J. Bot.*, **2017**, *49* (3), 1211–1216.

[18] Aziz, S. D. A.; Jafarah, N. F.; Sabri, S.; Wahab, M. A. A.; Yusof, Z. N. B. Antifungal Activities against Oil Palm Pathogen *Ganoderma Boninense* from Seaweed Sources. *Asia-Pacific J. Mol. Biol. Biotechnol.*, **2019**, *27* (1), 75–83. https://doi.org/10.35118/apjmbb.2019.027.1.08.

[19] Ambika, S.; Sujatha, K. Comparative Studies on Brown, Red and Green Alga Seaweed Extracts for Their Antifungal Activity against *Fusarium Oxysporum* f.Sp. *Udum* in Pigeon Pea Var. CO (Rg)7 (Cajanus Cajan (L.) Mills.). *J. Biopestic.*, **2014**, *7* (2), 167–176.

[20] Tyśkiewicz, K.; Tyśkiewicz, R.; Konkol, M.; Rój, E.; Jaroszuk-Ściseł, J.; Skalicka-Woźniak, K. Antifungal Properties of *Fucus Vesiculosus* L. Supercritical Fluid Extract Against *Fusarium Culmorum* and *Fusarium Oxysporum*. *Molecules*, **2019**, *24* (3518), 1–16.

[21] Mohamed, S. S.; Saber, A. A. Antifungal Potential of the Bioactive Constituents in Extracts of the Mostly Untapped Brown Seaweed *Hormophysa Cuneiformis* from The Egyptian Coastal Waters. *Egypt. J. Bot.*, **2019**, *59* (3), 695–708. https://doi.org/10.21608/ejbo.2019.5516.1225.

[22] Silva, P.; Fernandes, C.; Barros, L.; Ferreira, I. C. F. R.; Pereira, L.; Gonçalves, T. The Antifungal Activity of Extracts of Osmundea Pinnatifida, an Edible Seaweed, Indicates Its Usage as a Safe Environmental Fungicide or as a Food Additive Preventing Post-Harvest Fungal Food Contamination. *Food Funct.*, **2018**, *9*, 6187–6195. https://doi.org/10.1039/c8fo01797b.

[23] Gómez-Hernández, M.; Rodríguez-García, C. M.; Peraza-Echeverría, L.; Peraza-Sánchez, S. R.; Torres-Tapia, L. W.; Pérez-Brito, D.; Vargas-Coronado, R. F.; Cauich-Rodríguez, J. V. In Vitro Antifungal Activity Screening of Beach-Cast Seaweeds Collected in Yucatan, Mexico. *J. Appl. Phycol.*, **2021**.

[24] Zhang, Y.; Han, J.; Mu, J.; Feng, Y.; Gu, X.; Ji, Y. Bioactivity and Constituents of Several Common Seaweeds. *Chinese Sci. Bull.*, **2013**, *58* (19), 2282–2289. https://doi.org/10.1007/s11434-013-5745-y.

[25] Rizvi, M. A.; Shameel, M. Pharmaceutical Biology of Seaweeds from the Karachi Coast of Pakistan. *Pharm. Biol.*, **2005**, *43* (2), 97–107. https://doi.org/10.1080/13880200590919366.

[26] Ehteshamul-Haque, S.; Baloch, G. N.; Sultana, V.; Ara, J.; Tariq, R. M.; Athar, M. Impact of Seaweeds on Fluorescent Pseudomonas and Their Role in Suppressing the Root Diseases of Soybean and Pepper. *J. Appl. Bot. Food Qual.*, **2013**, *86*, 126–132. https://doi.org/10.5073/JABFQ.2013.086.017.

[27] Baloch, G. N.; Tariq, S.; Ehteshamul-Haque, S.; Athar, M.; Sultana, V.; Ara, J. Management of Root Diseases of Eggplant and Watermelon with the Application of Asafoetida and Seaweeds. *J. Appl. Bot. Food Qual.*, **2013**, *86*, 138–142. https://doi.org/10.5073/JABFQ.2013.086.019.

[28] Sultana, V.; Tariq, S.; Hira, K.; Tariq, A.; Ara, J.; Tariq, R. M.; Ehteshamul-Haque, S. Seaweed Bio-Fertilizer for the Management of Root Rotting Fungi and Root Knot Nematodes Affecting Cotton Crop. *Pakistan J. Bot.*, **2018**, *50* (December), 2409–2412.

[29] Sultana, V.; Baloch, G. N.; Ara, J.; Ehteshamul-haque, S.; Tariq, R. M.; Athar, M. Seaweeds as Alternative to Chemical Pesticides for the Management of Root Diseases of Sunflower and Tomato. *J. Appl. Bot. Food Qual.*, **2011**, *84*, 162–168.

[30] Sultana, V.; Ehteshamul-Haque, S.; Ara, J.; Athar, M. Effect of Brown Seaweeds and Pesticides on Root Rotting Fungi and Root-Knot Nematode Infecting Tomato Roots. *J. Appl. Bot. Food Qual.*, **2009**, *83*, 50–53.

[31] Sultana, V.; Baloch, G. N.; Ambreen; Ara, J.; Tariq, M. R.; Ehteshamul-Haque, S. Comparative Efficacy of a Red Alga Solieria Robusta, Chemical Fertilizers and Pesticides in Managing the Root Diseases and Growth of Soybean. *Pakistan J. Bot.*, **2011**, *43* (1), 1–6.
